# Nucleated Teleost Erythrocytes Play an Nk-Lysin- and Autophagy-Dependent Role in Antiviral Immunity

**DOI:** 10.3389/fimmu.2017.01458

**Published:** 2017-11-02

**Authors:** Patricia Pereiro, Alejandro Romero, Patricia Díaz-Rosales, Amparo Estepa, Antonio Figueras, Beatriz Novoa

**Affiliations:** ^1^Instituto de Investigaciones Marinas, Consejo Superior de Investigaciones Científicas (CSIC), Vigo, Spain; ^2^Instituto de Biología Molecular y Celular (IBMC), Universidad Miguel Hernández, Elche, Spain

**Keywords:** teleost, erythrocytes, red blood cells, autophagy, autophagolysosome, Nk-lysin, granulysin

## Abstract

With the exception of mammals, vertebrate erythrocytes are nucleated. Nevertheless, these cells are usually considered as mere carriers of hemoglobin. In this work, however, we describe for the first time an unrecognized role of teleost red blood cells (RBCs). We found that Nk-lysin (Nkl), an antimicrobial peptide produced by NK-cells and cytotoxic T-lymphocytes, was also expressed in flatfish turbot (*Scophthalmus maximus*) erythrocytes. Although the antiviral role of Nkl remains to be elucidated, we found a positive correlation between the transcription of *nkl* and the resistance to an infection with Rhabdovirus in a teleost fish. Surprisingly, Nkl was found to be present in the autophagolysosomes of erythrocytes, and therefore this higher resistance provided by Nkl could be related to autophagy. The organelles of RBCs are degraded through autophagy during the maturation process of these cells. In this work, we observed that the blockage of autophagy increased the replication of viral hemorrhagic septicemia virus in nucleated teleost erythrocytes, which suggests that this mechanism may also be a key process in the defense against viruses in these cells. Nkl, which possesses membrane-perturbing ability and was affected by this modulation of RBC autophagy, could also participate in this process. For the first time, autophagy has been described not only as a life cycle event during the maturation of erythrocytes but also as a pivotal antiviral mechanism in nucleated erythrocytes. These results suggest a role of erythrocytes and Nkl in the antiviral immunity of fish and other vertebrates with nucleated RBCs.

## Introduction

Erythrocytes, or red blood cells (RBCs), are the most abundant cells in the blood of vertebrates, and their primary function is to transport oxygen and carbon dioxide around the body. The enucleation of the erythroblast during erythropoiesis in mammals represents an evolutionary specialization that allows the increase of hemoglobin levels and enhances their flexibility and ability to traverse through capillaries (1, 2). Due to the anthropocentric vision that we unconsciously apply to our research, we may consider these cells as empty bags carrying hemoglobin without any other particular function. However, vertebrate evolution suggests that RBCs represent much more than that. Apart from mammals, vertebrate erythrocytes are nucleated and therefore possess the ability to modify their transcriptome and, in turn, their proteome. Any gene expressed even at low levels will achieve high proportions in the organism since RBCs are the most numerous blood cells in vertebrates. Little is known about the potential immune function of RBCs. Most of the investigations were carried out in mammals, and enucleated RBCs have only been implicated in some immune activities that are mainly mediated by hemoglobin (3–11). Although the information about the immune capabilities of nucleated erythrocytes is almost nonexistent, it was previously suggested that non-mammalian (fish and birds) erythrocytes possess the ability to specifically detect pathogen-associated molecular patterns (PAMPs) and participate in the immune response (12–16). In the case of teleost fish, there is still a lack of understanding of the immune function not only of erythrocytes but also of other immune cell populations such as natural killer and dendritic cells. This lack of understanding is mainly due to the absence of specific cell markers.

Red blood cells suffer a natural maturation process in which their organelles, such as mitochondria, endoplasmic reticulum, and peroxisomes, are degraded *via* autophagy (17, 18). In mammals, RBCs are released to the circulation in a complete mature stage; however, in teleost fish, a variable percentage of non-mature erythrocytes can be observed (19, 20). Autophagy is a highly conserved cellular self-degradative pathway in which cytoplasmic materials (e.g., misfolded or aggregated proteins, damaged organelles and/or intracellular pathogens) are engulfed into double-membrane bound vesicles for proteolytic degradation (21). This mechanism is also used by cells to obtain energy in response to starving conditions and during critical developmental processes (21). During the autophagy process, the autophagosome containing the cytoplasmic material for degradation fuses with a lysosome to form the autophagolysosome, where the lysosomal hydrolases degrade the enclosed materials (22). In addition to the role of the autophagy in the maintenance of the cell homeostasis, this process is implicated in the defense against intracellular pathogens, including viruses (23–25). Viral recognition by endosomal toll-like receptors (TLRs) or cytoplasmic viral nucleic-acid sensors can mediate the induction of autophagy for viral degradation in autophagolysosomes (26–29). Autophagy can also activate other innate and adaptive immune responses to fight against the virus (25). Moreover, autophagy is known to play a key role in the defense against Rhabdoviruses that affect both mammals and fishes (30–32). Nevertheless, this process has not been previously associated with the antiviral defense in erythrocytes.

In this work, we investigated the antimicrobial peptide (AMP) Nk-lysin (Nkl, orthologous to human granulysin), which has been considered to be produced by natural killer cells (NK-cells) and cytotoxic T lymphocytes (CTLs) and stored in cytolytic granules together with perforin and granzymes (33, 34). Surprisingly, Nkl was found in the autophagolysosomes of turbot RBCs. Our results also indicate that Nkl is involved in the resistance against viral hemorrhagic septicemia virus (VHSV) in turbot, and therefore, we hypothesize that autophagy might be the mechanism linking Nkl to VHSV resistance. Indeed, the blockage of autophagy in erythrocytes favored the viral replication in these cells and also affected the levels of Nkl. This suggests that fish erythrocytes play an active role against VHSV mediated through autophagy and involves Nkl. These data open the door to further investigations on the implication of erythrocytes and Nkl in the immunity of fish and other vertebrates with nucleated RBCs.

## Materials and Methods

### Characterization and Phylogenetic Analysis of Turbot Nkl

The complete open reading frame (ORF) of the turbot *nk-lysin* (*nkl*) gene was obtained from a previous 454-pyrosequencing of turbot tissues (35) and confirmed by sequencing using specific primers (Table S1 in Supplementary Material). A local blast against the turbot genome (36) was conducted to identify other potential *nkl* genes and to determine the number of exons/introns constituting the turbot *nkl*.

The presence of signal peptide was analyzed with the SignalP 3.0^1^ server (37) and the presence of specific domains with SMART 4.0^2^ (38). The three-dimensional (3D) structure of turbot Nkl was predicted using I-TASSER server (39) selecting the model with the best C-score and viewed by PyMOL.^3^ An alignment between several Nkls/granulysins protein sequences from fish, birds, and mammals was conducted using the ClustalW server (40). A phylogenetic tree was drawn using Mega 6.0 software (41) and selecting the model of protein evolution that best fits a given alignment according to the ProtTest 2.4^4^ server (42). Sequence similarity and identity scores were calculated with the software MatGAT (43) using the BLOSUM62 matrix. The GenBank accession numbers of the sequences used in this section are listed in Table S2 in Supplementary Material.

### Fish and Virus

Juvenile and adult turbot (average weight 2.5 and 125 g, respectively) were obtained from a commercial fish farm (Insuiña S.L., Galicia, Spain). Prior to experiments, fish were acclimatized to the laboratory conditions for 2 weeks. When necessary, fish were euthanized *via* MS-222 overdose (500 mg/L). All the experimental procedures were reviewed and approved by the CSIC National Committee on Bioethics under approval number ES360570202001/16/FUN01/PAT.05/tipoE/BNG.

Viral hemorrhagic septicemia virus (strain UK-860/94) was propagated in the Epithelioma Papulosum Cyprini (EPC) cell line (ATCC, CRL-2872) at 14°C in MEM (Gibco) supplemented with 2% FBS (Gibco), and 100 µg/mL Primocin (InvivoGen). The virus stock was titrated into 96-well plates according to established protocols (44, 45). VHSV aliquots were stored at −80°C until use.

### *nkl* Expression Plasmid, HEK-293 Cell Line, and Cell Transfection

The expression plasmid pMCV1.4-*nkl* was synthesized by ShineGene Molecular Biotech, Inc. (Shanghai, China) using the pMCV1.4 plasmid (Ready-Vector, Madrid, Spain) and the nucleotide sequences encoding the turbot Nkl mature peptide. The plasmid was cloned by transforming One Shot TOP10F’ competent cells (Invitrogen) and purified using the PureLink™ HiPure Plasmid Midiprep Kit (Invitrogen).

Human HEK-293 cells (ATCC CRL-1573) were grown in Eagle’s Minimum Essential Medium (Gibco) supplemented with 100 µg/mL primocin (InvivoGen), 1× non-essential amino acids (Gibco), 1-mM sodium pyruvate (Gibco), and 10% FBS. The cells were incubated in a 5% CO_2_ atmosphere at 37°C.

Recombinant Nkl was produced by transfection of 6 µg of the plasmid pMCV1.4-*nkl* into HEK-293 cells at 70–80% confluence (T-25 flask) using the XtremeGENE HP DNA Transfection Reagent (Roche) according to the manufacturer’s instructions. The same process was conducted with the corresponding empty plasmid pMCV1.4. Forty-eight hours after transfection, the supernatants were collected, filtered by 0.22 µm, and stored at −80°C until further use.

### Anti-Nkl Polyclonal Antibody Production and Validation

Emini surface accessibility scale (45), Kolaskar and Tongaonkar antigenicity scale (46), and Bepipred Linear Epitope Prediction (47) methods were used to predict the best Nkl antigen binding regions of antibodies. Based on this information, two peptides were chosen (RSLEINIDDQEQVC and CLFYPKQEESQTE). To obtain the anti-Nkl polyclonal antibody (New England Peptide, Gardner, MA, USA), rabbits were co-immunized with both synthetic peptides. Blood was collected before injection (pre-immune serum) and 30 days after the immunization (polyclonal antibody).

The anti-Nkl polyclonal antibody was validated by western blot (WB). For this, 15 µL of the supernatants from HEK-293 cells transfected with pMCV1.4-*nkl* or pMCV1.4 were mixed with 1× NuPAGE LDS Sample Buffer (Invitrogen) and resolved in a 4–20% Mini-PROTEAN TGX™ gel (Bio-Rad) (with and without 2-Mercaptoethanol and heat treatment 5 min at 95°C), and transferred to a nitrocellulose membrane (Bio-Rad). The membrane was blocked for 2 h with 3% (w/v) bovine serum albumin (BSA) in TBST buffer (20-mM Tris, 0.5-M NaCl, 0.1% Tween 20) and incubated for 2 h with the rabbit anti-Nkl polyclonal antibody (dilution 1:500 in 1% BSA-TBST buffer) at room temperature (RT). After three 10-min washes with TBST, membrane was incubated with a goat anti-rabbit-HRP antibody (Sigma) (dilution 1:10,000) for 1 h at RT, washed again, and revealed by chemiluminescence detection with Luminata™ Forte Western HRP Substrate (Millipore), and visualized with the ChemiDoc XRS + system (Bio-Rad).

### *nkl* Constitutive Expression in Different Tissues and *In Vivo* Induction after VHSV Challenge

To examine the constitutive expression of *nkl*, 11 different tissues (peritoneal exudate cells – PEC–, blood, head kidney, trunk kidney, spleen, gill, liver, intestine, heart, brain and muscle) were obtained from three adult healthy fish. PECs were obtained as previously described (48).

The modulation of *nkl* was also analyzed after an *in vivo* VHSV infection. A total of 50 juvenile turbot were divided into two groups. The first group (*n* = 25) was intraperitoneally (i.p.) injected with 50 µL of a VHSV suspension containing 2 × 10^6^ TCID_50_/mL. The second group was injected with the same volume of culture medium. The head kidney and spleen were removed from five turbot at 1, 2, 3, and 7 days post-infection (dpi), constituting five biological replicates for each tissue and sampling point. These samples were processed for the analysis of *nkl* expression.

### Correlation between *nkl* Transcription Level and Resistance to VHSV

*nkl* expression was analyzed in the head kidney samples from four turbot families showing different mortality rates after VHSV infection. Two VHSV-resistant (1 and 4) and two-susceptible families (2 and 3) were previously described by Diaz-Rosales et al. (49). Five animals of each family were analyzed before (naïve) and 24 h after the VHSV challenge conducted by Díaz-Rosales et al. (49). The expression of *nkl* was analyzed by quantitative polymerase chain reaction (qPCR).

Additionally, the correlation between the constitutive expression level of *nkl* gene in blood before a viral infection and the resistance to a VHSV challenge was determined. Approximately 20 µL of blood were extracted from the caudal vein of juvenile turbot using a heparinized syringe and cells were processed for the analysis of *nkl* expression. One week after the blood extraction, the turbot were i.p. injected with 100 µL of a VHSV suspension (3 × 10^7^ TCID_50_/mL). Mortality was recorded for 15 days and the size and weight of each turbot were also registered. The correlation between *nkl* mRNA levels in blood before the infection, the day of death, size and weight was determined using the Spearman’s rho correlation test.

### *nkl* Expression in Blood Cells and *In Vitro* VHSV Replication

The expression of *nkl* and the replication of VHSV in blood and in purified erythrocytes were analyzed by qPCR. Blood was taken from the caudal vein of three adult turbot using a heparinized syringe. Erythrocytes were purified in a Percoll (GE Healthcare) 51% gradient by centrifuging at 400× *g* for 30 min at 4°C without brake. Total blood cells and purified erythrocytes were adjusted to 10^8^ cells/mL in MEM (Gibco) supplemented with 2% FBS (Gibco) and 100 µg/mL Primocin (InvivoGen). Cells (250 µL) were distributed onto 24-well plates. A proportion of these cells were infected with VHSV (10^4^ TCID_50_/mL) and the remaining wells were maintained as controls. Cells were incubated at 15°C and collected 2, 3, 5, and 7 dpi for the quantification of *nkl* transcripts and VHSV glycoprotein gene by qPCR.

### Chloroquine (CQ) and Rapamycin Treatments

For qPCR analysis, erythrocytes were purified, seeded, infected with VHSV, and maintained as mentioned above. A proportion of the erythrocytes were incubated with CQ (25 µM; Sigma-C6628) or rapamycin (RAP) (5 µM; Sigma-R0395). Cell samples were taken 1, 2, and 3 dpi for the quantification of *nkl, becn1* and *atg5* transcripts and VHSV glycoprotein gene.

For confocal microscopy analysis, total blood cells were incubated with CQ (25 µM) or RAP (5 µM) for 3 days. Cells were fixed and immunostained as described below.

### Effect of the DNA Vaccine Encoding the VHSV Glycoprotein (pMCV1.4-G)

The modulation of the *nkl* gene and the protein abundance and distribution of Nkl under vaccination and/or infection conditions were evaluated by qPCR and flow cytometry. Twelve adult turbot were intramuscularly (i.m.) injected with 50 µL of a DNA vaccine (2 µg/fish) encoding the G glycoprotein from VHSV (pMCV1.4-G_860_) (50), whereas the other 12 fish were injected with the same amount of the empty plasmid (pMCV1.4). One month after vaccination, 6 fish from each group were i.p. injected with 50 µL of VHSV (2 × 10^6^ TCID_50_/mL) and the remaining six fish were injected with cell culture medium. The head kidney, spleen, and blood were taken at 48 h post-infection (hpi). Cell suspensions from the head kidney and spleen were prepared by passing the tissue through a 40-µm nylon mesh in phosphate-buffered saline (PBS). Blood was collected from the caudal vein using a heparinized syringe and diluted in PBS. All the samples were divided into two groups to be analyzed at the same time by qPCR and flow cytometry.

### RNA Extraction, cDNA Synthesis, and Real-time qPCR Analysis

Total RNA from the different tissue samples was extracted using the Maxwell^®^ 16 LEV simplyRNA Tissue kit (Promega) with the automated Maxwell^®^ 16 Instrument in accordance with instructions provided by the manufacturer. The cDNA synthesis was performed with the SuperScript II Reverse Transcriptase (Invitrogen) using 0.5 µg of RNA and following the manufacturer indications, except for the blood cell samples, in which case the cDNA synthesis was conducted with SuperScript III Reverse transcriptase (Invitrogen) using 0.1 µg of RNA.

Gene expression profiles were determined using real-time qPCR. Specific qPCR primers were designed using the Primer3 program (51) and their amplification efficiency was calculated using seven, fivefold serial dilutions of cDNA from unstimulated turbot with the threshold cycle (CT) slope method (52). The identity of the amplicon was confirmed by sequencing. Individual qPCR reactions were conducted in 25-µL reaction volume using 12.5 µL of SYBR GREEN PCR Master Mix (Applied Biosystems), 10.5 µL of ultrapure water (Sigma-Aldrich), 0.5 µL of each specific primer (10 µM), and 1 µL of fivefold diluted cDNA template in MicroAmp optical 96-well reaction plates (Applied Biosystems). All reactions were performed using technical triplicates in a 7300 Real-Time PCR System thermocycler (Applied Biosystems) with an initial denaturation (95°C, 10 min) followed by 40 cycles of a denaturation step (95°C, 15 s) and one hybridization–elongation step (60°C, 1 min). No-template controls were also included on each plate to detect possible contamination or primer dimers formed during the reaction. An analysis of melting curves was performed for each reaction. Relative expression of each gene was normalized using the eukaryotic translation *elongation factor 1 alpha* (*ef1a*) as reference gene, which was constitutively expressed and not affected by the experimental treatments, and was calculated using the Pfaffl method (52). Primer sequences used for the quantification of *nkl, becn1, atg5*, and VHSV glycoprotein transcripts are listed in Table S1 in Supplementary Material.

### Immunofluorescence Assays, Flow Cytometry, and Confocal Microscopy

The head kidney, spleen, total blood cells, and purified erythrocytes samples were obtained as previously described from adult turbots. Cells were fixed with 2% paraformaldehyde during 15 min at 4°C. After washing, the cells were blocked by incubating for 1 h in PBS with 0.1% saponin (Sigma) and 2% of BSA (Sigma). Then, cells were incubated overnight at 4°C with the preimmune serum or with the rabbit anti-Nkl polyclonal antibody in staining buffer (PBS with 0.1% saponin and 0.1% BSA) (dilution 1:250). Cells were then washed and incubated with the secondary antibody Alexa Fluor^®^ 488 goat anti-rabbit IgG (Molecular Probes-Life Technologies; 1:1,000) for 1 h at RT. Samples were washed and resuspended in PBS. The expression of Nkl was analyzed using a FACSCalibur flow cytometer (BD Biosciences) in dot plots of relative size (forward-light-scatter, FSC) and complexity (side-light-scatter, SSC) in linear and logarithmic scale. FL1-H histograms were used to compare the fluorescence levels emitted by samples labeled with the anti-Nkl antibody and the preimmune serum. The percentage of positive fluorescent events and the intensity of fluorescence (median) were registered.

Cells were adjusted to 10^6^ cells/mL and distributed onto 24-well plates with 12-mm glass coverslips and incubated at 15°C for 2 h before the fixation. Cells were fixed with 2% paraformaldehyde for 15 min at 4°C. After washing, the cells were blocked by incubating for 1 h in PBS with 0.1% saponin (Sigma) and 2% of BSA (Sigma). Then, cells were incubated overnight at 4°C with the corresponding primary antibody in staining buffer (PBS with 0.1% saponin and 0.1% BSA). Cells were then washed and incubated with the secondary antibody for 1 h at RT. Nkl was stained using the rabbit anti-Nkl polyclonal antibody (1:250) and the secondary antibody Alexa Fluor^®^ 488 goat anti-rabbit IgG or Alexa Fluor^®^ 546 goat anti-rabbit IgG (Molecular Probes-Life Technologies) (1:1,000), depending on the experiment. Autophagy activity was analyzed using a rabbit anti-LC3A/B polyclonal antibody (Cell Signaling; 4108S) (1:200), or a mouse anti-LC3B monoclonal antibody (Nanotools; 0231-100/LC3-5F10) (1:20) for co-localization assays. The Alexa Fluor^®^ 546 goat anti-rabbit IgG and Alexa Fluor^®^ 488 goat anti-mouse IgG (1:1,000) were used as secondary antibodies, respectively. The immune detection of VHSV in blood samples was performed using the mouse anti-N VHSV monoclonal antibody 3E7 (1:1,000) (53) and the secondary antibody Alexa Fluor^®^ 635 goat anti-mouse IgG (Molecular Probes-Life Technologies) (1:1,000). All samples were stained with a DAPI solution (Molecular Probes-Life Technologies) for nuclear localization and mounted using ProLong Antifade Reagents (Life Technologies). LysoSensor blue DND-167 reagent (Molecular Probes-Life Technologies) was used to stain the acidic lysosomal vesicles in live cells. Confocal images were captured using a TSC SPE confocal microscope (Leica) using the LAS AF software (Leica). The 3D reconstructions were performed using the Image Surfer^5^ software.

### Transmission Electron Microscopy (TEM) Images

Blood samples were fixed overnight with 2% glutaraldehyde in 0.1-M cacodylate buffer at pH 7.4. Then, samples were washed and incubated with 1% tannic acid in 0.1-M cacodylate buffer at 4°C for 1 h and washed again. The cells were centrifuged at 1,000× *g* for 5 min and included in 1% agarose blocks, which were sectioned in 1 mm^3^ pieces and washed with 0.1-M cacodylate buffer. The sections were incubated for 1 h with 1% osmium tetroxide at 4°C and washed and dehydrated in increasing concentrations of ethanol. After the dehydration, samples were embedded in Epon resin and 65–85 nm sections were prepared using the ultramicrotome and mounted on metal grids. Ultrathin sections were stained with 50% uranyl acetate in methanol and lead citrate prior to observation with the JEOL JEM-1010 transmission electron microscope (Electron Microscopy Unit of CACTI, University of Vigo, Spain).

### Statistical Analysis

Both qPCR expression results and flow-cytometry fluorescence data were represented graphically as the mean/median + the standard deviation of the biological replicates. To determine significant differences, data were analyzed with the computer software package SPSS v.19.0 using the Student’s *t*-test or ANOVA as appropriate. For the correlation analysis, Spearman’s Rho correlation coefficient was calculated. Differences were considered statistically significant at *p* < 0.05.

## Results

### Turbot *nkl*

**Figure 1:**
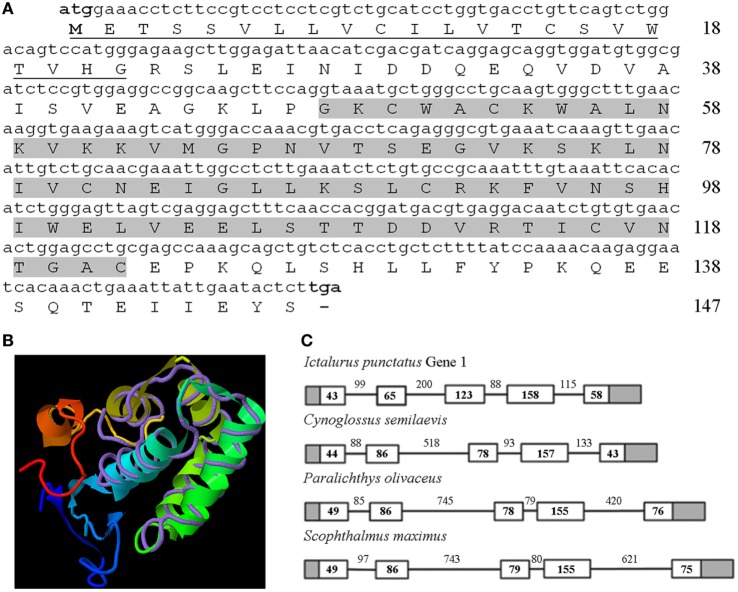
Characterization of turbot Nkl. **(A)** The complete coding region of turbot *nkl* (444-bp long) encodes a protein of 147 residues. The signal peptide is underlined and the SapB domain is highlighted. **(B)** 3D structure of turbot Nkl using the pig protein as a template (TM score = 0.582). The tertiary structure comprises six α-helices. **(C)** Structure of the turbot *nkl* gene and alignment between the coding region and the corresponding genomic sequence. This gene contains five exons and four introns. The 5′ and 3′ UTRs are represented as gray boxes, the CDSs of the exons as white boxes, and introns as solid lines. The length (bp) of the CDSs and introns is also reflected in the figure. CDSs, coding DNA sequences; Nkl, Nk-lysin; SapB, saposin B; UTRs, untranslated regions.

The complete coding region of the turbot *nkl* gene was deposited in GenBank under Acc. No. KU705506. The characteristic saposin B (SapB) domain of the saposin-like proteins (SAPLIP) family was identified (Figure 1A). The 3D structure was constructed with a moderate confidence value using Nkl from pig as a template (TM score = 0.582) (Figure 1B). On the other hand, the gene structure (exon/intron organization) was conserved among teleost fish (Figure 1C) and compared with other vertebrates.

A multiple alignment of several Nkls/granulysin amino-acid sequences from fish, birds, and mammals revealed six cysteine residues that were well conserved among the different species (Figure S1A in Supplementary Material). A phylogenetic tree showed two main clusters, one of them containing teleost Nkl and the other one containing avian and mammalian sequences (Figure S1B in Supplementary Material). As expected, an identity/similarity matrix (Table S3 in Supplementary Material) revealed that turbot Nkl shares the highest scores with the other flatfish species, but when it was compared with sequences from birds and mammals, the identity ranked between 16 and 20% and the similarity between 36 and 45%.

### *nkl* Transcription Level Related to Antiviral Response

**Figure 2:**
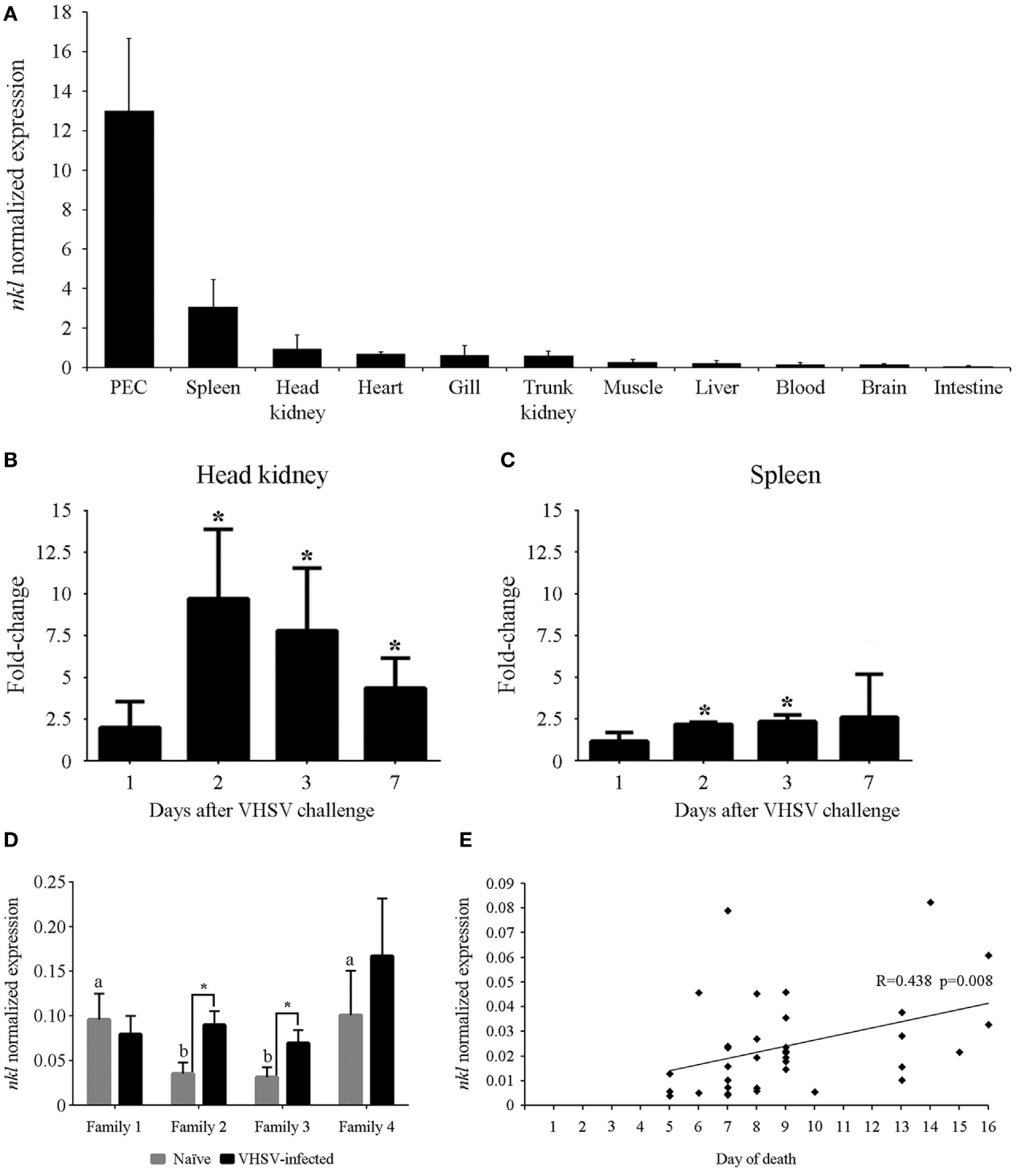
Tissue distribution of the *nkl* expression, induction after a VHSV challenge in lymphoid tissues, and relation between nkl transcription and resistance against VHSV. **(A)** Constitutive expression of *nkl* in different tissues from healthy adult turbot. Normalized expression values are represented as the mean of three individuals plus SD. **(B,C)** Modulation of *nkl* gene expression in the head kidney **(C)** and spleen **(B)** after VHSV infection. Data are expressed as fold-change regarding the values obtained in controls at each sampling point. Graphs represent the mean of five biological replicates plus SD. Significant overexpression (*p* < 0.05) are represented with asterisks. **(D)** Normalized expression of *nkl* gene in head kidney samples from resistant families (1 and 4) and susceptible families (2 and 3), before and after VHSV infection. Graph represents the mean of five biological replicates plus SD. Letters **(A,B)** indicate significant differences (*p* < 0.05) between the families in naïve conditions. Asterisks indicate significant differences (*p* < 0.05) between the naïve and VHSV-infected condition in each family. **(E)** Correlation between *nkl* transcription level in blood cells (before infection) and the day of death after VHSV challenge in juvenile turbot. The Spearman’s Rho correlation test showed a significant (*p* = 0.008) but moderate (*R* = 0.438) positive correlation between both variables. VHSV, viral hemorrhagic septicemia virus.

The constitutive expression of the *nkl* gene was determined in different tissues from healthy turbot. *nkl* transcription was detected in all the tested tissues but the highest expression levels were detected in immune tissues (peritoneal exudate cells –PEC–followed by the spleen and head kidney) (Figure 2A). When turbot were infected with VHSV, the *nkl* gene was overexpressed in the two main immune organs in fish, head kidney (Figure 2B) and spleen (Figure 2C), suggesting an antiviral response.

The expression of *nkl* was analyzed in head kidney samples from fish belonging to VHSV-resistant and VHSV-susceptible families, before (naïve) and after VHSV challenge. The resistant families (1 and 4) showed a significantly higher expression of *nkl* than the susceptible ones (2 and 3) before the viral infection. Interestingly, 24 h after VHSV challenge, a significant increase in *nkl* transcription was only observed in susceptible families (2 and 3) (Figure 2D).

We used a non-destructive method (blood sampling from caudal vein) to determine the *nkl* transcription level before infection in 35 turbot and, after VHSV challenge, individual mortality was registered. The correlation between *nkl* mRNA level in blood cells, turbot size and weight, and the day of death after VHSV infection was analyzed using the Spearman’s rho correlation test. Mortalities started 4 days after infection, reaching 90% cumulative mortality at the end of the experiment (15 days after infection). A significant positive correlation between *nkl* constitutive transcription in the blood and the day of death was observed (*R* = 0.438; *p* = 0.008) (Figure 2E): turbot with higher constitutive expression of *nkl* survived longer after infection than those with low expression level. Turbot size and weight were also correlated with the day of death, but no relationship between size or weight and the level of *nkl* transcripts was detected (Table S4 in Supplementary Material). Therefore, the basal transcription of *nkl* is a size/weight-independent factor, and its level is correlated with the resistance to VHSV infection.

### VHSV Glycoprotein Inducing Long-Lasting Effects in the Levels of Nkl

**Figure 3:**
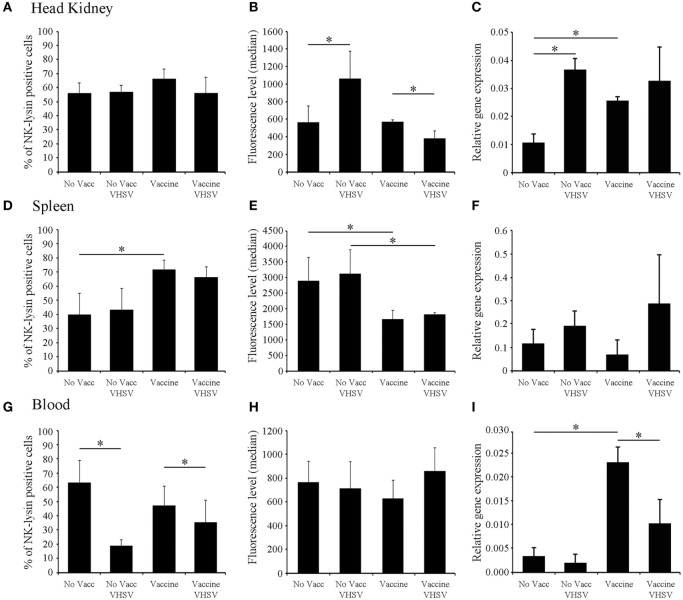
Modulation of Nkl during vaccination and/or infection in different tissues by flow cytometry and qPCR. **(A–C)** head kidney; **(D–F)**, spleen; **(G–I)**, blood. **(A,D,G)** represent the percentage of Nkl-positive cells in the three tissues. **(B,E,H)** represent the median value of the intensity of fluorescence in positive cells in the three tissues. **(C,F,I)** represent the expression of *nkl* in the tissues by qPCR. The positive region in the FL1-H histogram was adjusted using the signal registered in the same samples stained with the preimmune serum. Gating values for the positive region were 300-9910 for the head kidney, 514-9910 for spleen, and 262-9910 for blood samples. In all figures, the mean value of six samples plus SD is represented. Asterisks indicate significant differences (*p* < 0.05) between experimental groups. Nkl, Nk-lysin; qPCR, quantitative polymerase chain reaction.

The expression levels of *nkl* in the head kidney, spleen, and blood were analyzed by qPCR 1 month after the injection of a highly efficient DNA vaccine encoding the VHSV glycoprotein (pMCV1.4-G) (50) or the corresponding control empty plasmid (pMCV1.4) under both healthy and VHSV-infected conditions (48 hpi) (Figure 3). In parallel, flow-cytometry analysis of cell populations from the three different tissues was also conducted (Figure 3). Surprisingly, 1 month after vaccination, there was an increase in the transcription of the *nkl* gene detected in the head kidney and blood samples (Figures 3C,I), but not in the spleen (Figure 3F). Nevertheless, after a VHSV challenge, the expression of *nkl* increased only in the head kidney from non-vaccinated individuals but decreased in blood samples from vaccinated fish (Figures 3C,I). To detect the presence of Nkl at the protein level, we designed and used an anti-Nkl polyclonal antibody. After confirming the specificity of the antibody by WB (Figure S2 in Supplementary Material), flow-cytometry analysis was conducted. Differences among vaccinated and non-vaccinated turbot (in the absence of infection) were only observed in the spleen, with an increase in the percentage of Nkl-positive cells but a reduction in the median value of fluorescence per cell (Figures 3D,E). After the VHSV challenge, Nkl increased in the head kidney from non-vaccinated fish but decreased in vaccinated turbot (Figure 3B); however, there were no differences in the number of Nkl-positive cells (Figure 3A). In the blood, the percentage of Nkl-positive cells decreased after infection in both vaccinated and non-vaccinated fish (Figure 3G), but no significant differences in the level of Nkl were detected in these cells (Figure 3H).

### Nkl Distribution in the Head Kidney and Blood Cells

**Figure 4:**
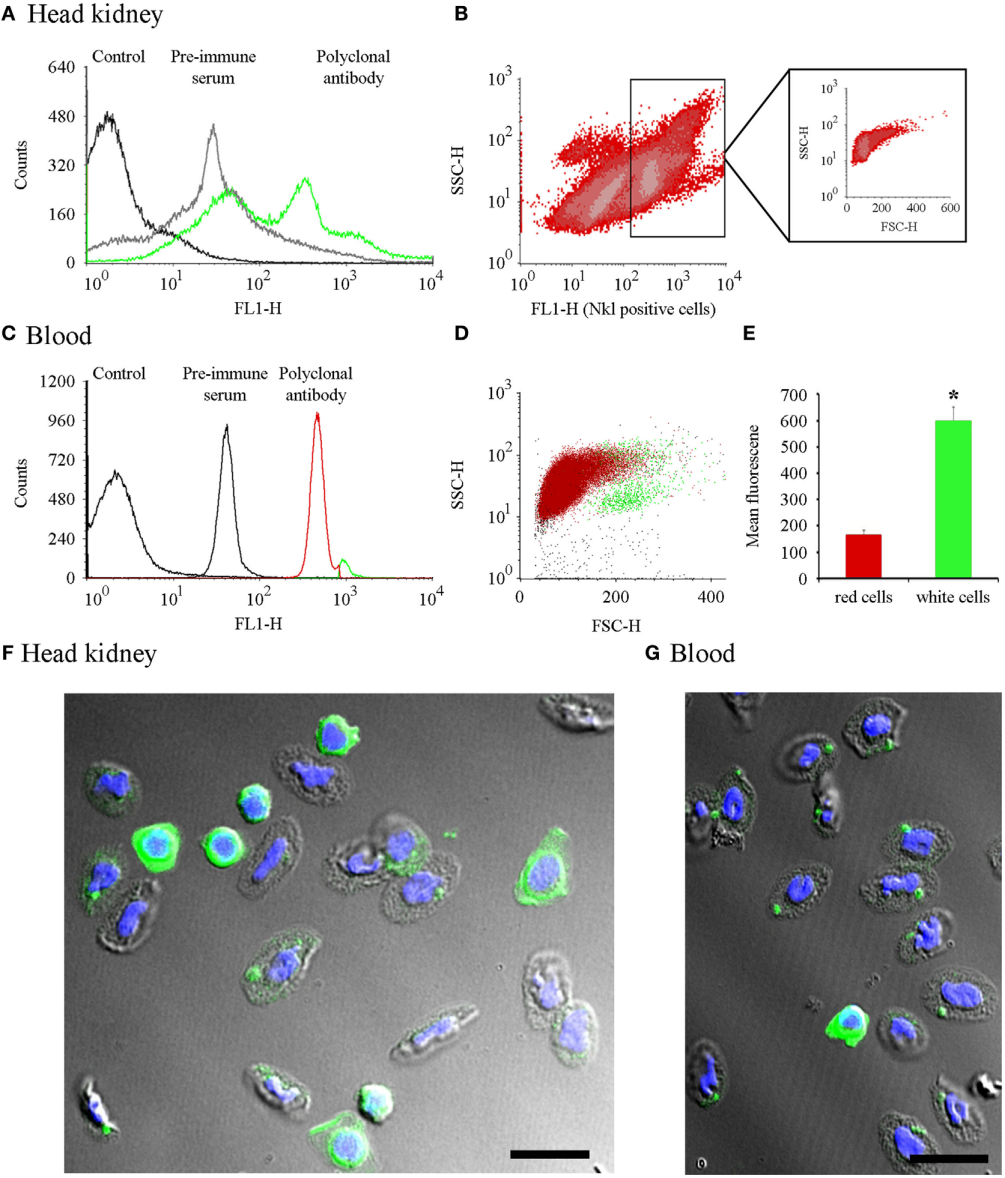
Cell distribution of Nkl by flow cytometry in head kidney and blood samples and confocal images of cells stained with the anti-Nkl antibody. **(A)** A FL1-H histogram was used to compare the fluorescence profile of head kidney samples stained with the preimmune serum or the polyclonal antibody. FL1-H positive cells were gating at 300-9910. **(B)** Distribution of head kidney cell populations using SSC-H vs. FL1-H density plots. The FSC-H/SSC-H position of Nkl-positive stained cells is boxed. **(C)** Fluorescence profile of blood samples stained with preimmune serum and polyclonal antibody. Positive cells for Nkl were gated at 262-9910. **(D)** Dot plot representing the position of the two Nkl-positive cell populations (red: cell population enriched in erythrocytes or red cells; green: cell population enriched in white cells). **(E)** Mean fluorescence value registered in blood cell populations. Asterisks indicate significant differences (*p* < 0.05). **(F,G)** Confocal microscopy images of a head kidney and a blood sample, respectively, showing white and red cells stained with the anti-Nkl polyclonal antibody. Green: Nkl. Blue; DAPI. Scale bar, 10 µm. FSC, forward-light-scatter; Nkl, Nk-lysin; SSC, side-light-scatter.

Flow cytometry was conducted on head kidney cells and total blood samples (Figures 4A,C). Nkl-positive cells gated in the FL1-H histogram were represented in FSC/SSC density plots (Figures 4B,D). In the head kidney, the positive cells were clustered in a heterogeneous population showing low size and complexity (Figure 4B). In blood samples, the fluorescence histogram revealed the presence of two clear populations (Figure 4C) with different positions in the FSC/SSC density plot (Figure 4D). One population corresponded to cells with the lowest fluorescence level and small size and complexity. Erythrocytes are the most abundant cell type in this population. The other population consisted of cells that had a higher fluorescence, were larger in size, and were essentially the white cell population. A clear significant difference in the fluorescence level was detected among both populations. Fluorescence values for erythrocytes were 168 ± 16.5 and 605 ± 52.4 for leukocytes (Figure 4E).

To visualize the expression of Nkl in these cells, the distribution of the Nkl peptide was analyzed by confocal microscopy of the head kidney and blood cells. In the small spherical cells with a high nucleus/cytoplasm ratio, probably corresponding to CTLs and the hypothetical NK-cells, the cytoplasm was completely stained with the anti-Nkl antibody (Figures 4F,G). Turbot erythrocytes were also positive for Nkl-immunostaining (Figures 4F,G).

### *nkl* Expression in Erythrocytes

**Figure 5:**
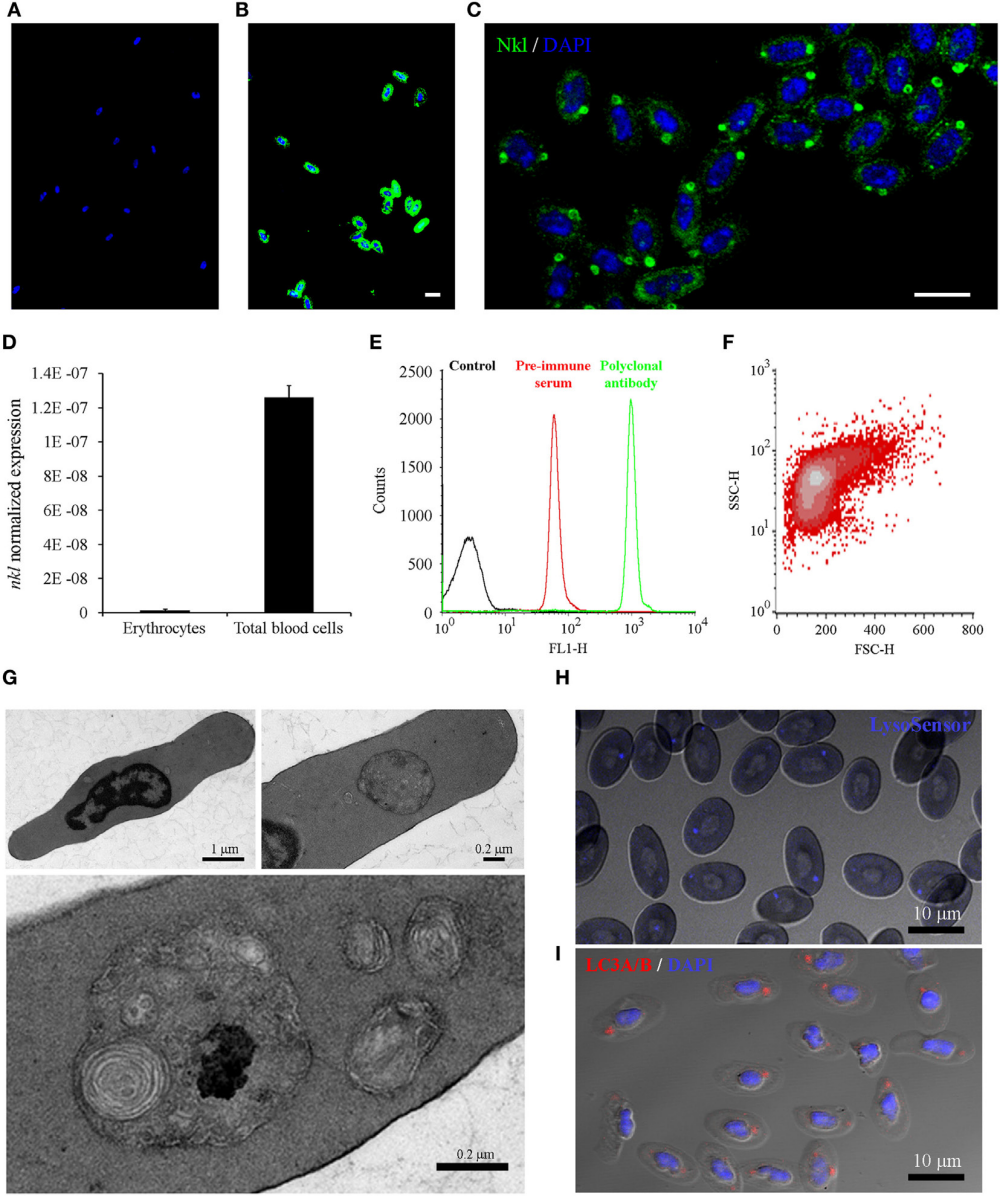
Analysis of Nkl in erythrocytes by confocal microscopy, qPCR and flow cytometry, and the characterization of the cytoplasmic structures containing Nkl. **(A,B)** Immunocytofluorescence of purified erythrocytes stained with the rabbit preimmune serum **(A)** or the anti-Nkl polyclonal antibody **(B)**, and the secondary antibody Alexa Fluor 488 goat anti-rabbit IgG. Nuclei were stained with DAPI. Scale bar, 10 µm. The nonspecific fluorescence of the preimmune serum was not registered in samples stained with the polyclonal antibody. **(C)** Nkl was mainly detected in a large, spherical structure. Moreover, small puncta were also present in the cytoplasm. Scale bar, 10 µm. **(D)** Normalized expression of *nkl* in total blood cells and in purified erythrocytes. Bars represents the mean plus SD of 3 individual samples. **(E)** FL1-H fluorescence profile of purified erythrocytes stained with preimmune serum and polyclonal antibody. A specific signal of the antibody was clearly registered. **(F)** FSC/SSC density plot of FL1-H positive cells gated between 262-9910 log scale. **(G)** Description of the Nkl-positive vesicles by TEM. Almost all turbot erythrocytes present a large, double-membrane bound structure in the cytoplasm, although a few smaller ones are also observed **(H)** LysoSensor Blue staining of turbot erythrocytes. The spherical structures containing Nkl are LysoSensor-positive acidic vesicles. Scale bar, 10 µm. **(I)** Immunocytofluorescence of purified erythrocytes stained with the rabbit anti-LC3A/B polyclonal antibody, and the Alexa Fluor 546 goat anti-rabbit IgG as secondary antibody. Nuclei were stained with DAPI. Scale bar, 10 µm. Nkl, Nk-lysin; qPCR, quantitative polymerase chain reaction; TEM, transmission electron microscopy.

The unexpected presence of Nkl in erythrocytes was analyzed in detail. Confocal images of erythrocytes immunostained with the preimmune serum or the anti-Nkl antibody confirmed the specificity of the polyclonal antibody (Figures 5A,B). Almost all erythrocytes were found to be Nkl-positive and this peptide was mainly expressed in a large, spherical cytoplasmic structure; however, a few small Nkl-positive spots were also observed (Figure 5C). The mRNA expression of *nkl* in this cell type was lower compared with that of total blood cells (Figure 5D), suggesting that the expression of this gene is much higher in other *nkl*-expressing cells present in blood (leukocytes). The FL1-H fluorescence profile of purified erythrocytes showed a well-defined peak (Figure 5E) corresponding to a homogeneous cell population (Figure 5F).

To further characterize the structure of the cytoplasmic vesicles, a study using Transmission electron microscopy (TEM) was conducted (Figure 5G). At low magnification, a large, spherical cytoplasmic structure was observed in almost all erythrocytes, although in some cells other smaller vesicles were also found. These structures showed a double-membrane surrounding electron-dense structures, which probably correspond to cellular organelles such as mitochondria, Golgi apparatus, and endoplasmic reticulum. LysoSensor staining revealed the acidic nature of these spherical structures in the cytoplasm of erythrocytes (Figure 5H), and immunostaining with a rabbit polyclonal anti-LC3 antibody showed that autophagy is also occurring in these structures (Figure 5I). These data suggested that these Nkl-containing structures probably correspond to autophagolysosomes.

### Nkl Involvement in Autophagy

To fully elucidate whether these Nkl-positive vesicles correspond to the LC3-positive structures, we conducted a co-localization analysis after *in vitro* stimulations of erythrocytes with the autophagy inhibitor CQ and the autophagy activator RAP. The execution of autophagy involves the participation of numerous proteins; however, during the final steps of autophagy, only microtubule-associated protein light chain-3 (LC3) is known to exist in mature autophagolysosomes (54).

**Figure 6:**
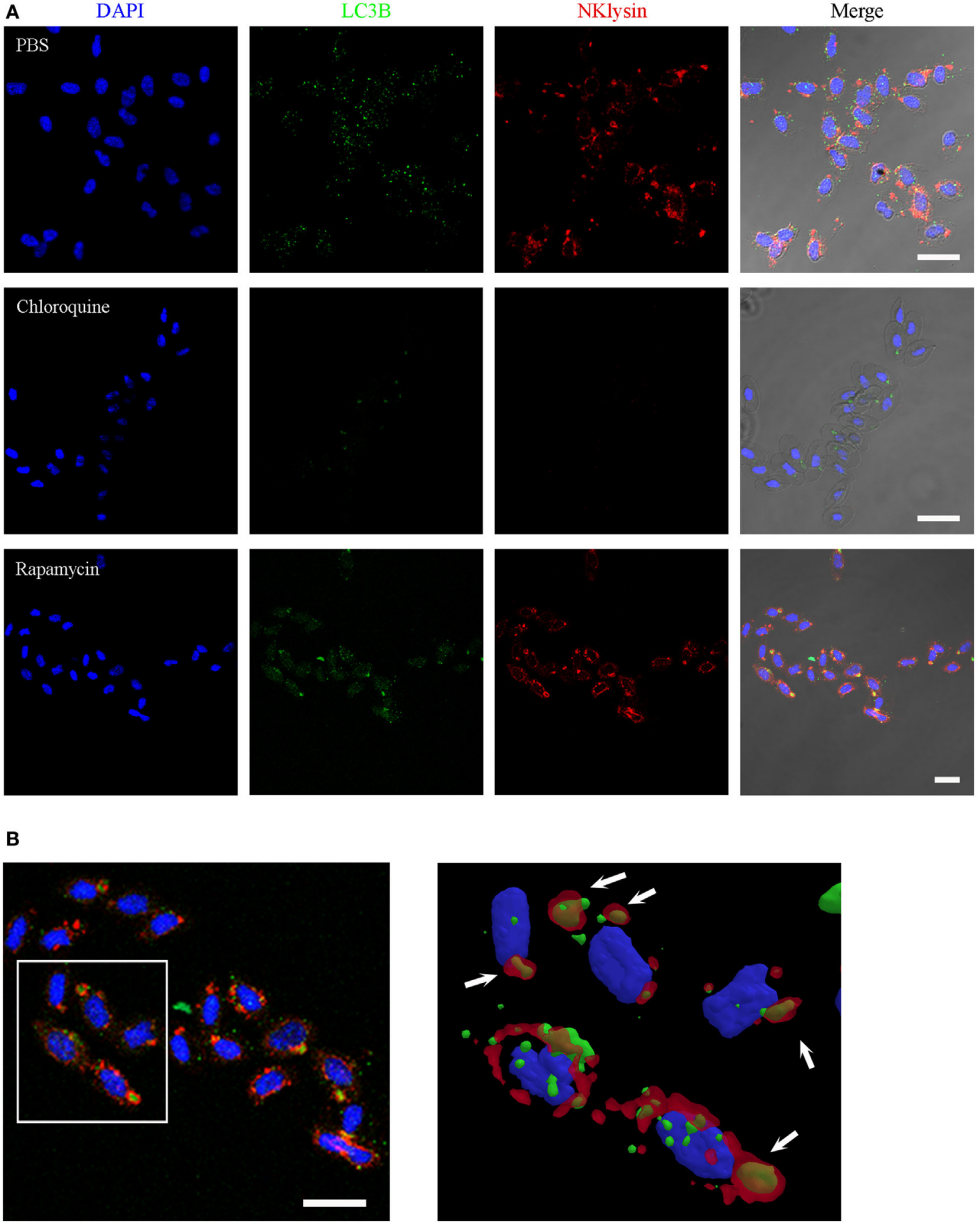
Nkl associated with autophagolysosomes in erythrocytes. **(A)** Immunofluorescence confocal images of turbot erythrocytes in the absence of treatment (PBS) or after incubation with chloroquine or rapamycin. The cells were immunostained with anti-Nkl and anti-LC3B antibodies (Alexa Fluor 546 goat anti-rabbit IgG and Alexa Fluor 488 goat anti-mouse IgG as secondary antibodies, respectively). Nuclei were stained with DAPI. Merged images showed co-localization of Nkl and LC3B in the erythrocytes incubated with rapamycin. Red: Nkl, Green: LC3B, Blue: DAPI. Scale bar, 10 µm. **(B)** 3D reconstruction of confocal images of erythrocytes incubated with rapamycin showing the double-positive (Nkl and LC3B) autophagolysosomes (white arrows). Nkl, Nk-lysin; PBS, phosphate-buffered saline.

Although the spherical Nkl-positive structures were also labeled with the rabbit polyclonal anti-LC3 A/B antibody (Figure 5I), in the co-localization studies, the mouse monoclonal anti-LC3B antibody resulted in LC3B-positive punctate structures that were dispersed in the cytoplasm of untreated erythrocytes (Figure 6A) whereas Nkl was strongly detected in the autophagolysosomes. Therefore, LC3B and Nkl did not co-localize. Interestingly, the incubation of the erythrocytes with CQ and RAP completely modified this pattern after 24 h. In the CQ-treated cells, LC3B was now confined to the autophagolysosomes, and the Nkl signal disappeared (Figure 6A). This is probably because CQ raises intravesicular pH (55) and the SAPLIP show markedly increased activities at acidic pH (56) because of their pH-dependent conformational properties (57). On the other hand, the autophagy activator RAP also affected the distribution of LC3B. In this case, although numerous LC3B-positive puncta were also observed in the cytoplasm, the higher fluorescent signal was found in the autophagolysosomes where a strong co-localization of LC3B and Nkl was detected (Figure 6A). A detailed 3D reconstruction of the erythrocytes incubated with RAP showed that Nkl seems to surround the LC3B signal in the autophagic structures (Figure 6B).

### Erythrocytes Showing Antiviral Activity That Depends on Autophagy and Nkl

**Figure 7:**
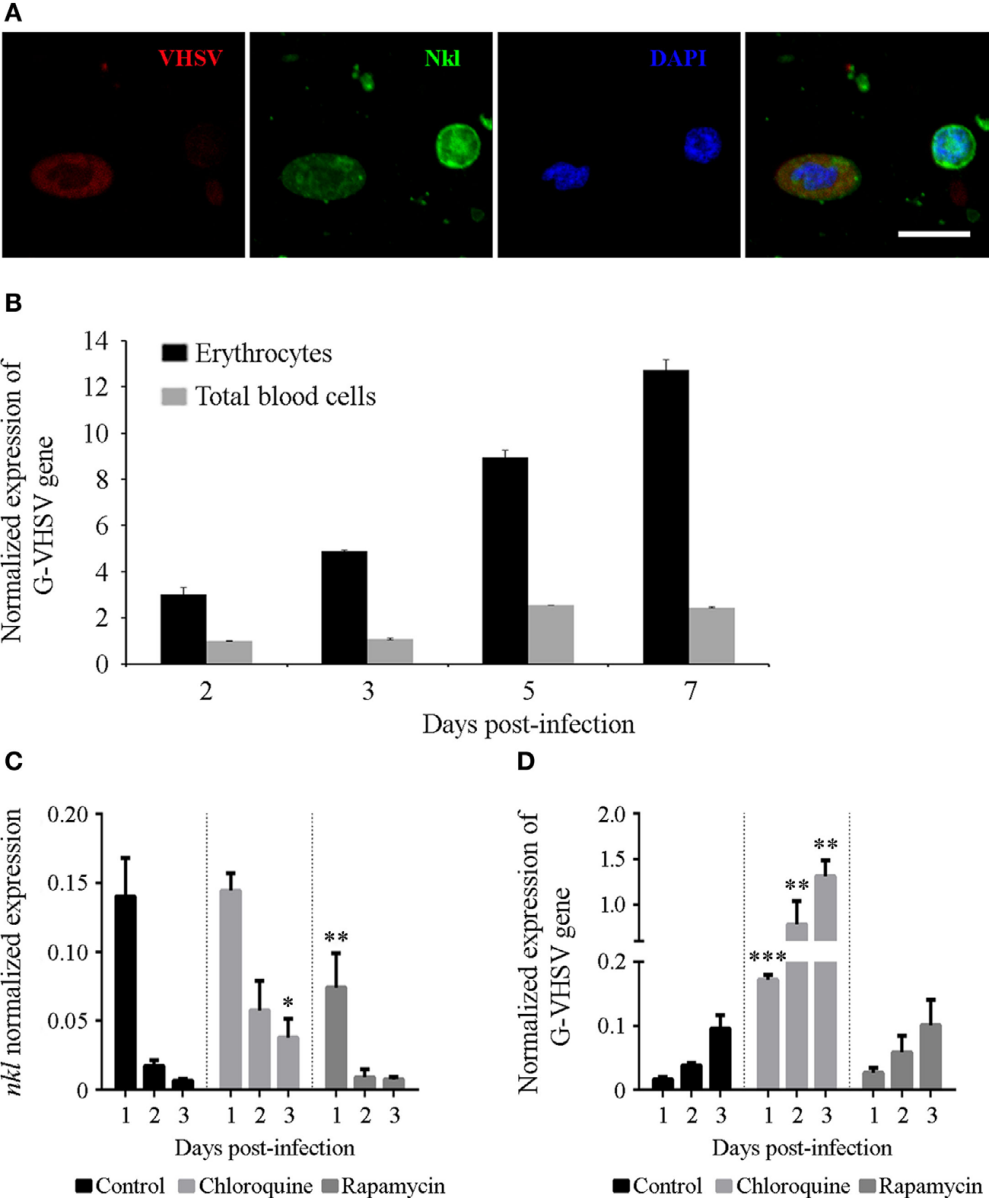
VHSV infecting and replicating in erythrocytes. **(A)** Confocal images of double-immunofluorescence staining of VHSV nucleoprotein −N− and Nkl in blood cells. The virus was stained with the mouse anti-N VHSV monoclonal antibody 3E7 and the secondary antibody Alexa Fluor 635 goat anti-mouse IgG, and Nkl with the rabbit anti-Nkl polyclonal antibody and Alexa Fluor 488 goat anti-rabbit IgG. Nuclei were stained with DAPI. Red: N-VHSV, Green: Nkl, Blue: DAPI. Scale bar, 10 µm. **(B)** The replication of VHSV in erythrocytes and total blood cells after an *in vitro* infection was measured by qPCR detection of the VHSV glycoprotein −G− gene. Bars represents the mean plus SD of three individual samples. **(C,D)** Effect of chloroquine and rapamycin in the transcription of *nkl* **(C)** and in the VHSV replication **(D)** in erythrocytes. Bars represents the mean plus SD of three individual samples. Nkl, Nk-lysin; qPCR, quantitative polymerase chain reaction; VHSV, viral hemorrhagic septicemia virus.

*In vitro* infection of total blood cells and erythrocytes revealed another interesting finding. Erythrocytes were found to be positive for VHSV infection using immunofluorescence staining (Figure 7A), although flow cytometry revealed that VHSV-positive erythrocytes were less than 1% after 24 h. VHSV replication was analyzed both in total blood cells and erythrocytes by qPCR detection of the viral glycoprotein (G). Viral replication was higher in erythrocytes compared with total blood cells, and the viral detection increased over time (Figure 7B). Opposed to the VHSV replication, the time-course experiment revealed that, both in total blood cells and erythrocytes, the transcription of *nkl* decreases in a time-dependent, infection-independent manner (Figures S3A,B in Supplementary Material).

The effect of CQ and RAP during *in vitro* VHSV infection in erythrocytes was also studied. CQ and RAP modulated the mRNA levels of autophagy-related genes (Figures S3C,D in Supplementary Material) and, interestingly, the substances affected the transcription of *nkl* in an opposite manner. CQ treatment damped the time-dependent reduction of *nkl* transcription, whereas RAP significantly reduced the level of *nkl* transcription after 24 h (Figure 7C).

While there were no significant differences in the detection of the VHSV G gene in those erythrocytes incubated with RAP compared with the control, autophagy blockage with CQ favored viral replication, which is reflected through the higher detection of the VHSV G gene in the cells incubated with this compound (Figure 7D). This result indicates that autophagy is an important antiviral defense mechanism in these cells.

## Discussion

Nk-lysin is an AMP involved in the destruction of bacteria, fungi and parasites. Nevertheless, only a few publications suggest a potential role of Nkl in the antiviral immune response, probably because an Nkl/granulysin ortholog gene has not been identified in the mouse or rat model species (58). Nk-lysin was found to be overexpressed after viral challenge in chicken (59, 60) and fish (61–63), and some evidence of its antiviral effect were reported (63–65). In this work, we observed that turbot *nkl* transcription is positively correlated with VHSV resistance. We conducted numerous *in vivo* and *in vitro* experiments both using an expression plasmid encoding Nkl (pMCV1.4-*nkl*) or the recombinant protein, but we did not obtain any significant difference in mortality or viral replication after an infection with VHSV. Therefore, we were not able to provide any evidence of direct antiviral activity. This is probably due to the fact that Nkl does not work as a typical AMP in the antiviral context, and it needs to be confined into the autophagolysosomes.

Although Nkl was always assumed to be present only in the cytolytic granules of NK-cells and CTLs (33, 34), this does not seem to be the case in teleost fish. Flow-cytometry analysis and confocal microscopy unexpectedly revealed that this peptide is expressed in turbot erythrocytes. In these cells, fluorescence was confined to one or to a few vesicles in the cytoplasm, which were also LysoSensor- and LC3B-positive. TEM images revealed that these structures are double-membrane bound compartments containing cellular organelles. These data confirmed that these vesicles correspond to autophagolysosomes. Is Nkl contributing to the self-degradation of the RBCs organelles as a part of the maturation process? Nk-lysin, as a member of the SAPLIP family, possesses membrane-perturbing ability (66), and therefore it could be intervening in the disruption of the biological membranes of cellular organelles. Moreover, other members of the SAPLIP family, such as the saposin peptides, are important in the correct resolution of autophagy due to their specific roles in the degradation of the glycosphingolipids present in biological membranes (67, 68). Except for mammals, vertebrate erythrocytes are nucleated. During the first embryonic stages in mammals a transient population of nucleated erythrocytes is present in the bloodstream, whereas in late fetal periods and postnatal life only enucleated RBCs are found in the circulating blood (69). This early presence of nucleated RBCs in embryos could represent an evolutionary reminiscence from non-mammalian vertebrates.

Autophagy is also an important cellular mechanism in the clearance of viruses (23–29) and represents a key process in the defense against Rhabdoviruses in mammals and fish (30–32). Nevertheless, in erythrocytes, autophagy has been always relegated to function in the degradation of cellular organelles during the maturation of these cells. In turbot, it was previously reported that VHSV, which is a highly pathogenic virus affecting turbot (70), can infect primary cultures of blood leukocytes (71). However, in this work we observed that VHSV has also the ability to infect and proliferate in erythrocytes during an *in vitro* infection. Inhibition of the autophagic process using CQ increased the viral replication, indicating that autophagy is an important antiviral mechanism in RBCs. Nevertheless, we should take into consideration that CQ also affects the antigen presentation *via* major histocompatibility complex (MHC) class I and class II (72, 73), although the relevance of this process during an *in vitro* infection in erythrocytes is probably negligible.

Currently, autophagy is an emerging field of study and, although the main components of the autophagy machinery are well known, only a few articles have examined the relation between AMPs and autophagy. These publications are mainly focused on the generation of neo-AMPs with bactericidal activity from cytosolic proteins in the mycobacteria-containing autophagolysosomes (74–76). Ren et al. (77) found that a peptide derived from the human cathelicidin could activate caspase-independent apoptosis and autophagy in colon cancer cells. Interestingly, it was also reported that human granulysin induces the cleavage of Atg5 in the complex formed with Atg12 although no effects in autophagy were observed (78).

It seems that Rhabdovirus-induced autophagy is mediated, at least in part, by the viral G glycoprotein (30, 32, 79). In this work, we observed that a DNA vaccine encoding the VHSV G glycoprotein (50) induces long-lasting effects on the levels of Nkl. Because autophagy is an important mechanism in the generation of innate memory cells (80, 81), the persistence of changes in the levels of Nkl 1 month after vaccination could indicate that this peptide is also important in the “trained immunity” or innate memory. Therefore, autophagy could be the process linking the levels of Nkl and the resistance to VHSV. Nevertheless, more investigation is needed to fully understand how Nkl levels determine the antiviral state of teleost fish and if erythrocytes actively contribute to this process.

Two major conclusions can be extracted from our work: (A) Teleost erythrocytes have an active antiviral role that is mediated by autophagy and (B) this is the first time that an AMP, Nkl, is associated with the autophagic mechanism. The results that support these conclusions have been unrecognized until now: the correlation between *nkl* expression and the resistance to viruses; the presence of Nkl in nucleated fish erythrocytes; the relation between Nkl and autophagy; and the implication of autophagy in the antiviral response of erythrocytes. Taken together, these results can change the preconceived ideas that we have about vertebrate immunity, opening new doors to combat diseases that, in the case of fish, seriously affect the aquaculture industry and focusing more on cells and processes previously not considered.

## Ethics Statement

All the experimental procedures were reviewed and approved by the CSIC National Committee on Bioethics (Protocol no. ES360570202001/16/FUN01/PAT.05/tipoE/BNG).

## Author Contributions

AE, AF, and BN conceived and designed the study, and analyzed the data. PP, AR, and PD-R performed the experimental procedures and data analyses. PP, AR, AF, and BN wrote the manuscript. All the authors reviewed the manuscript.

## Conflict of Interest Statement

The authors declare that the research was conducted in the absence of any commercial or financial relationships that could be construed as a potential conflict of interest.

## Footnotes

^1^http://www.cbs.dtu.dk/services/SignalP/.

^2^http://smart.embl.de/.

^3^http://www.pymol.org.

^4^http://darwin.uvigo.es/software/prottest2_server.html.

^5^http://cismm.web.unc.edu/software/.

## References

[1] Ji P Murata-Hori M Lodish HF. Formation of mammalian erythrocytes: chromatin condensation and enucleation. Trends Cell Biol (2011) 21:409–15. 10.1016/j.tcb.2011.04.003 21592797 PMC3134284

[2] Keerthivasan G Wickrema A Crispino JD. Erythroblast enucleation. Stem Cells Int (2011) 2011:139851. 10.4061/2011/139851 22007239 PMC3189604

[3] Antunes RF Brandão C Maia M Arosa FA. Red blood cells release factors with growth and survival bioactivities for normal and leukemic T cells. Immunol Cell Biol (2011) 89:111–21. 10.1038/icb.2010.60 20440295

[4] Arosa FA Pereira CF Fonseca AM. Red blood cells as modulators of T cell growth and survival. Curr Pharm Des (2004) 10:191–201. 10.2174/1381612043453432 14754398

[5] Buttari B Profumo E Riganò R. Crosstalk between red blood cells and the immune system and its impact on atherosclerosis. Biomed Res Int (2015) 2015:616834. 10.1155/2015/616834 25722984 PMC4334626

[6] Fonseca AM Porto G Uchida K Arosa FA. Red blood cells inhibit activation-induced cell death and oxidative stress in human peripheral blood T lymphocytes. Blood (2001) 97:3152–60. 10.1182/blood.V97.10.3152 11342443

[7] Jiang N Tan NS Ho B Ding JL. Respiratory protein-generated reactive oxygen species as an antimicrobial strategy. Nat Immunol (2007) 8:1114–22. 10.1038/ni1501 17721536

[8] Kawano T Pinontoan R Hosoya H Muto S. Monoamine-dependent production of reactive oxygen species catalyzed by pseudoperoxidase activity of human hemoglobin. Biosci Biotechnol Biochem (2002) 66:1224–32. 10.1271/bbb.66.1224 12162542

[9] Liepke C Baxmann S Heine C Breithaupt N Ständker L Forssmann WF. Human hemoglobin-derived peptides exhibit antimicrobial activity: a class of host defense peptides. J Chromatogr B Analyt Techno Biomed Life Sci (2003) 791:345–56. 10.1016/S1570-0232(03)00245-9 12798194

[10] Schäkel K von Kietzell M Hänsel A Ebling A Schulze L Haase M Human 6-sulfo LacNAc-expressing dendritic cells are principal producers of early interleukin-12 and are controlled by erythrocytes. Immunity (2006) 24:767–77. 10.1016/j.immuni.2006.03.020 16782032

[11] Zhang Q Xu Y Wang Q Hang B Sun Y Wei X Potential of novel antimicrobial peptide P3 from bovine erythrocytes and its analogs to disrupt bacterial membranes *in vitro* and display activity against drug-resistant bacteria in a mouse model. Antimicrob Agents Chemother (2015) 59:2835–41. 10.1128/AAC.04932-14 25753638 PMC4394822

[12] Morera D Roher N Ribas L Balasch JC Doñate C Callol A RNA-Seq reveals an integrated immune response in nucleated erythrocytes. PLoS One (2011) 6:e26998. 10.1371/journal.pone.0026998 22046430 PMC3203173

[13] Morera D MacKenzie SA. Is there a direct role for erythrocytes in the immune response? Vet Res (2011) 42:89. 10.1186/1297-9716-42-89 21801407 PMC3199785

[14] Passantino L Altamura M Cianciotta A Patruno R Tafaro A Jirillo E Fish immunology. I. Binding and engulfment of *Candida albicans* by erythrocytes of rainbow trout (*Salmo gairdneri* Richardson). Immunopharmacol Immunotoxicol (2002) 24:665–78. 10.1081/IPH-120016050 12510797

[15] Passantino L Massaro MA Jirillo F Di Modugno D Ribaud MR Modugno GD Antigenically activated avian erythrocytes release cytokine-like factors: a conserved phylogenetic function discovered in fish. Immunopharmacol Immunotoxicol (2007) 29:141–52. 10.1080/08923970701284664 17464774

[16] Workenhe ST Kibenge MJ Wright GM Wadowska DW Groman DB Kibenge FS. Infectious salmon anaemia virus replication and induction of alpha interferon in Atlantic salmon erythrocytes. Virol J (2008) 5:36. 10.1186/1743-422X-5-36 18307775 PMC2292172

[17] Betin VMS Singleton BK Parsons SF Anstee DJ Lane JD. Autophagy facilitates organelle clearance during differentiation of human erythroblasts: evidence for a role for ATG4 paralogs during autophagosome maturation. Autophagy (2013) 9:881–93. 10.4161/auto.24172 23508006 PMC3672297

[18] Zhang J Wu K Xiao X Liao J Hu Q Chen H Autophagy as a regulatory component of erythropoiesis. Int J Mol Sci (2015) 16:4083–94. 10.3390/ijms16024083 25689426 PMC4346945

[19] Glomski CA Tamburlin J Chainani M. The phylogenetic odyssey of the erythrocyte. III. Fish, the lower vertebrate experience. Histol Histopathol (1992) 7:501–28. 1504472

[20] Tavares-Dias M A morphological and cytochemical study of erythrocytes, thrombocytes and leukocytes in four freshwater teleosts. J Fish Biol (2006) 68:1822–33. 10.1111/j.1095-8649.2006.01089.x

[21] Glick D Barth S Macleod KF. Autophagy: cellular and molecular mechanisms. J Pathol (2010) 221:3–12. 10.1002/path.2697 20225336 PMC2990190

[22] Mizushima N Yoshimori T Levine B. Methods in mammalian autophagy research. Cell (2010) 140:313–26. 10.1016/j.cell.2010.01.028 20144757 PMC2852113

[23] Levine B Kroemer G. Autophagy in the pathogenesis of disease. Cell (2008) 132:27–42. 10.1016/j.cell.2007.12.018 18191218 PMC2696814

[24] Mizushima N Levine B Cuervo AM Klionsky DJ. Autophagy fights disease through cellular self-digestion. Nature (2008) 451:1069–75. 10.1038/nature06639 18305538 PMC2670399

[25] Shoji-Kawata S Levine B. Autophagy, antiviral immunity, and viral countermeasures. Biochim Biophys Acta (2009) 1793:1478–84. 10.1016/j.bbamcr.2009.02.008 19264100 PMC2739265

[26] Berryman S Brooks E Burman A Hawes P Roberts R Netherton C Foot-and-mouth disease virus induces autophagosomes during cell entry via a class III phosphatidylinositol 3-kinase-independent pathway. J Virol (2012) 86:12940–53. 10.1128/JVI.00846-12 22993157 PMC3497631

[27] Delgado MA Elmaoued RA Davis AS Kyei G Deretic V Toll-like receptors control autophagy. EMBO J (2008) 27:1110–21. 10.1038/emboj.2008.31 18337753 PMC2323261

[28] McFarlane S Aitken J Sutherland JS Nicholl MJ Preston VG Preston CM. Early induction of autophagy in human fibroblasts after infection with human cytomegalovirus or herpes simplex virus 1. J Virol (2011) 85:4212–21. 10.1128/JVI.02435-10 21325419 PMC3126239

[29] Shi CS Kehrl JH. MyD88 and Trif Target Beclin 1 to trigger autophagy in macrophages. J Biol Chem (2008) 283:33175–82. 10.1074/jbc.M804478200 18772134 PMC2586260

[30] García-Valtanen P Ortega-Villaizán M Martínez-López A Medina-Gali R Pérez L Mackenzie S Autophagy-inducing peptides from mammalian VSV and fish VHSV rhabdoviral G glycoproteins (G) as models for the development of new therapeutic molecules. Autophagy (2014) 10:1666–80. 10.4161/auto.29557 25046110 PMC4206542

[31] Nakamoto M Moy RH Xu J Bambina S Yasunaga A Shelly SS Virus recognition by Toll-7 activates antiviral autophagy in *Drosophila*. Immunity (2012) 36:658–67. 10.1016/j.immuni.2012.03.003 22464169 PMC3334418

[32] Shelly S Lukinova N Bambina S Berman A Cherry S. Autophagy is an essential component of *Drosophila* immunity against vesicular stomatitis virus. Immunity (2009) 30:588–98. 10.1016/j.immuni.2009.02.009 19362021 PMC2754303

[33] Andersson M Gunne H Agerberth B Boman A Bergman T Sillard R NK-lysin, a novel effector peptide of cytotoxic T and NK cells. Structure and cDNA cloning of the porcine form, induction by interleukin 2, antibacterial and antitumour activity. EMBO J (1995) 14:1615–25. 7737114 10.1002/j.1460-2075.1995.tb07150.x PMC398254

[34] Peña SV Krensky AM. Granulysin, a new human cytolytic granule-associated protein with possible involvement in cell-mediated cytotoxicity. Semin Immunol (1997) 9:117–25. 10.1006/smim.1997.0061 9194222

[35] Pereiro P Balseiro P Romero A Dios S Forn-Cuni G Fuste B High-throughput sequence analysis of turbot (*Scophthalmus maximus*) transcriptome using 454-pyrosequencing for the discovery of antiviral immune genes. PLoS One (2012) 7:e35369 10.1371/journal.pone.0035369 22629298 PMC3356354

[36] Figueras A Robledo D Corvelo A Hermida M Pereiro P Rubiolo JA Whole genome sequencing of turbot (*Scophthalmus max*imus; Pleuronectiformes): a fish adapted to demersal life. DNA Res (2016) 23:181–92. 10.1093/dnares/dsw007 26951068 PMC4909306

[37] Emanuelsson O Brunak S von Heijne G Nielsen H. Locating proteins in the cell using TargetP, SignalP and related tools. Nat Protoc (2007) 2:953–71. 10.1038/nprot.2007.131 17446895

[38] Letunic I Copley RR Schmidt S Ciccarelli FD Doerks T Schultz J SMART 4.0: towards genomic data integration. Nucleic Acids Res (2004) 32:D142–4. 10.1093/nar/gkh088 14681379 PMC308822

[39] Roy A Kucukural A Zhang Y. I-TASSER: a unified platform for automated protein structure and function prediction. Nat Protoc (2010) 5:725–38. 10.1038/nprot.2010.5 20360767 PMC2849174

[40] Thompson JD Higgins DG Gibson TJ CLUSTAL W: improving the sensitivity of progressive multiple sequence alignments through sequence weighting, position specific gap penalties and weight matrix choice. Nucleic Acid Res (1994) 22:4673–80. 10.1093/nar/22.22.4673 7984417 PMC308517

[41] Tamura K Stecher G Peterson D Filipski A Kumar S. MEGA6: molecular evolutionary genetics analysis version 6.0. Mol Biol Evol (2013) 30:2725–9. 10.1093/molbev/mst197 24132122 PMC3840312

[42] Abascal F Zardoya R Posada D. ProtTest: selection of best-fit models of protein evolution. Bioinformatics (2005) 21:2104–5. 10.1093/bioinformatics/bti263 15647292

[43] Campanella JJ Bitincka L Smalley J MatGAT: an application that generates similarity/identity matrices using protein or DNA sequences. BMC Bioinform (2003) 4:29 10.1186/1471-2105-4-29 PMC166169 12854978

[44] Reed LJ Muench H Simple method of estimating fifty per cent endpoints. Am J Hyg (1938) 27:493–7. 10.1093/oxfordjournals.aje.a118408

[45] Emini EA Hughes JV Perlow DS Boger J Induction of hepatitis a virus-neutralizing antibody by a virus-specific synthetic peptide. J Virol (1985) 55:836–9. 2991600 10.1128/jvi.55.3.836-839.1985 PMC255070

[46] Kolaskar AS Tongaonkar PC A semi-empirical method for prediction of antigenic determinants on protein antigens. FEBS Lett (1990) 276:172–4. 10.1016/0014-5793(90)80535-Q 1702393

[47] Larsen JEP Lund O Nielsen M Improved method for predicting linear B-cell epitopes. Immunome Res (2006) 2:2 10.1186/1745-7580-2-2 16635264 PMC1479323

[48] Pereiro P Forn-Cuni G Figueras A Novoa B Pathogen-dependent role of turbot (*Scophthalmus maximus*) interferon-gamma. Fish Shellfish Immunol (2016) 59:25–35. 10.1016/j.fsi.2016.10.021 27742586

[49] Díaz-Rosales P Romero A Balseiro P Dios S Novoa B Figueras A. Microarray-based identification of differentially expressed genes in families of turbot (*Scophthalmus maximus*) after infection with viral haemorrhagic septicaemia virus (VHSV). Mar Biotechnol (NY) (2012) 14:515–29. 10.1007/s10126-012-9465-0 22790792

[50] Pereiro P Martinez-Lopez A Falco A Dios S Figueras A Coll JM Protection and antibody response induced by intramuscular DNA vaccine encoding for viral haemorrhagic septicaemia virus (VHSV) G glycoprotein in turbot (*Scophthalmus maximus*). Fish Shellfish Immunol (2012) 32:1088–94. 10.1016/j.fsi.2012.03.004 22554577

[51] Rozen S Skaletsky HJ Primer3 on the WWW for general users and for biologist programmers. In: Krawetz S Misener S, editors. Bioinformatics Methods and Protocols: Methods in Molecular Biology. Totowa, NJ: Humana Press (2000). p. 365–86. 10.1385/1-59259-192-2:365 10547847

[52] Pfaffl MW. A new mathematical model for relative quantification in real-time RT-PCR. Nucleic Acids Res (2001) 29:e45. 10.1093/nar/29.9.e45 11328886 PMC55695

[53] Sanz F Coll JM Detection of viral haemorrhagic septicemia virus by direct immunoperoxidase with selected anti-nucleoprotein monoclonal antibody. Bull Eur Assoc Fish Pathol (1992) 12:116–9.

[54] Mizushima N Methods for monitoring autophagy. Int J Biochem Cell Biol (2004) 36:2491–502. 10.1016/j.biocel.2004.02.005 15325587

[55] Steinman RM Mellman IS Muller WA Cohn ZA Endocytosis and the recycling of plasma membrane. J Cell Biol (1983) 96:1–27. 10.1083/jcb.96.1.1 6298247 PMC2112240

[56] Bruhn H. A short guided tour through functional and structural features of saposin-like proteins. Biochem J (2005) 389:249–57. 10.1042/BJ20050051 15992358 PMC1175101

[57] Vaccaro AM Ciaffoni F Tatti M Salvioli R Barca A Tognozzi D pH-dependent conformational properties of saposins and their interactions with phospholipid membranes. J Biol Chem (1995) 270:30576–80. 10.1074/jbc.270.51.30576 8530492

[58] Clayberger C Finn MW Wang T Saini R Wilson C Barr VA 15 kDa granulysin causes differentiation of monocytes to dendritic cells but lacks cytotoxic activity. J Immunol (2012) 188:6119–26. 10.4049/jimmunol.1200570 22586033 PMC3370151

[59] Sarson AJ Abdul-Careem MF Read LR Brisbin JT Sharif S Expression of cytotoxicity-associated genes in Marek’s disease virus-infected chickens. Viral Immunol (2008) 21:267–72. 10.1089/vim.2007.0094 18570592

[60] Chen CY Xie QM Xue Y Ji J Chang S Ma JY Characterization of cytotoxicity-related gene expression in response to virulent Marek’s disease virus infection in the bursa of Fabricius. Res Vet Sci (2013) 94:496–503. 10.1016/j.rvsc.2012.10.014 23164636

[61] Pereiro P Dios S Boltaña S Coll J Estepa A Mackenzie S Transcriptome profiles associated to VHSV infection or DNA vaccination in turbot (*Scophthalmus maximus*). PLoS One (2014) 9:e104509. 10.1371/journal.pone.0104509 25098168 PMC4123995

[62] Pereiro P Varela M Diaz-Rosales P Romero A Dios S Figueras A Zebrafish Nk-lysins: first insights about their cellular and functional diversification. Dev Comp Immunol (2015) 51:148–59. 10.1016/j.dci.2015.03.009 25813149

[63] Zhang M Long H Sun L. A NK-lysin from *Cynoglossus semilaevis* enhances antimicrobial defense against bacterial and viral pathogens. Dev Comp Immunol (2013) 40:258–65. 10.1016/j.dci.2013.03.005 23524198

[64] Hata A Zerboni L Sommer M Kaspar AA Clayberger C Krensky AM Granulysin blocks replication of varicella-zoster virus and triggers apoptosis of infected cells. Viral Immunol (2001) 14:125–33. 10.1089/088282401750234501 11398808

[65] Zhang M Li MF Sun L. NKLP27: a teleost NK-lysin peptide that modulates immune response, induces degradation of bacterial DNA, and inhibits bacterial and viral infection. PLoS One (2014) 9:e106543. 10.1371/journal.pone.0106543 25180858 PMC4152322

[66] Ruysschaert JM Goormaghtigh E Homblé F Andersson M Liepinsh E Otting G. Lipid membrane binding of NK-lysin. FEBS Lett (1998) 425:341–4. 10.1016/S0014-5793(98)00261-0 9559676

[67] Sun Y Zamzow M Ran H Zhang W Quinn B Barnes S Tissue-specific effects of saposin A and saposin B on glycosphingolipid degradation in mutant mice. Hum Mol Genet (2013) 22:2435–50. 10.1093/hmg/ddt096 23446636 PMC3708521

[68] Tatti M Motta M Di Bartolomeo S Cianfanelli V Salvioli R. Cathepsin-mediated regulation of autophagy in saposin C deficiency. Autophagy (2013) 9:241–3. 10.4161/auto.22557 23108186 PMC3552889

[69] Maximow A Untersuchungen über Blut und Bindegewebe. Die frühesten Entwicklungsstadien der Blut- und Bindegewebszellen beim Säugetierembryo, bis zum Anfang der Blutbildung in der Leber. Arch Mikrosk Anat (1908) 73:444–561. 10.1007/BF02979896

[70] Pereiro P Figueras A Novoa B Turbot (*Scophthalmus maximus*) vs VHSV (viral hemorrhagic septicemia virus): a review. Front Physiol (2016) 7:192 10.3389/fphys.2016.00192 27303308 PMC4880558

[71] Tafalla C Figueras A Novoa B. *In vitro* interaction of viral haemorrhagic septicaemia virus and leukocytes from trout (*Oncorhynchus mykiss*) and turbot (*Scophthalmus maximus*). Vet Immunol Immunopathol (1998) 62:359–66. 10.1016/S0165-2427(97)00167-0 9646440

[72] Ziegler HK Unanue ER Decrease in macrophage antigen catabolism caused by ammonia and chloroquine is associated with inhibition of antigen presentation to T cells. Proc Natl Acad Sci U S A (1982) 79:175–8. 10.1073/pnas.79.1.175 6798568 PMC345685

[73] Schultz KR Bader S Paquet J Li W. Chloroquine treatment affects T-cell priming to minor histocompatibility antigens and graft-versus-host disease. Blood (1995) 86:4344–52. 7492796

[74] Alonso S Pethe K Russell DG Purdy GE. Lysosomal killing of *Mycobacterium* mediated by ubiquitin-derived peptides is enhanced by autophagy. Proc Natl Acad Sci U S A (2007) 104:6031–6. 10.1073/pnas.0700036104 17389386 PMC1851611

[75] Kim BH Shenoy AR Kumar P Das R Tiwari S MacMicking JD. A family of IFN-γ-inducible 65-kD GTPases protects against bacterial infection. Science (2011) 332:717–21. 10.1126/science.1201711 21551061

[76] Ponpuak M Davis AS Roberts EA Delgado MA Dinkins C Zhao Z Delivery of cytosolic components by autophagic adaptor protein p62 endows autophagosomes with unique antimicrobial properties. Immunity (2010) 32:329–41. 10.1016/j.immuni.2010.02.009 20206555 PMC2846977

[77] Ren SX Shen J Cheng AS Lu L Chan RL Li ZJ FK-16 derived from the anticancer peptide LL-37 induces caspase-independent apoptosis and autophagic cell death in colon cancer cells. PLoS One (2013) 8:e63641. 10.1371/journal.pone.0063641 23700428 PMC3659029

[78] Aporta A Catalán E Galán-Malo P Ramírez-Labrada A Pérez M Azaceta G Granulysin induces apoptotic cell death and cleavage of the autophagy regulator Atg5 in human hematological tumors. Biochem Pharmacol (2014) 87:410–23. 10.1016/j.bcp.2013.11.004 24269628

[79] Denizot M Varbanov M Espert L Robert-Hebmann V Sagnier S Garcia E HIV-1 gp41 fusogenic function triggers autophagy in uninfected cells. Autophagy (2008) 4:998–1008. 10.4161/auto.6880 18818518

[80] Buffen K Oosting M Quintin J Ng A Kleinnijenhuis J Kumar V Autophagy controls BCG-induced trained immunity and the response to intravesical BCG therapy for bladder cancer. PLoS Pathog (2014) 10:e1004485. 10.1371/journal.ppat.1004485 25356988 PMC4214925

[81] Xu X Araki K Li S Han JH Ye L Tan WG Autophagy is essential for effector CD8(+) T cell survival and memory formation. Nat Immunol (2014) 15:1152–61. 10.1038/ni.3025 25362489 PMC4232981

