# Supplementary material for: Nucleated Teleost Erythrocytes Play an Nk-Lysin- and Autophagy-Dependent Role in Antiviral Immunity
Source: Front Immunol. 2017 Nov 2;8:1458. doi: 10.3389/fimmu.2017.01458 (PMC5673852; doi:10.3389/fimmu.2017.01458)

SUPPLEMENTARY MATERIAL

Nucleated teleost erythrocytes play an Nk-lysin- and autophagy-dependent role in antiviral immunity

Patricia Pereiro[1,#], Alejandro Romero[1,#], Patricia Díaz-Rosales[1], Amparo Estepa[2], Antonio Figueras[1,*], Beatriz Novoa[1,*]

[1]Instituto de Investigaciones Marinas, Consejo Superior de Investigaciones Científicas (CSIC), Vigo, Spain

[2]Instituto de Biología Molecular y Celular (IBMC), Universidad Miguel Hernández, Elche, Spain

Supplementary Table S1. Primer sequences used in open reading frame (ORF) confirmation and qPCR analysis.

Primer	Sequence	Application
nkl* ORF-F**<br>nkl* ORF-R**	ATGGAAACCTCTTCCGTCCT<br>GCATACAACATCAAGAGTATTCA	ORF confirmation
nkl* qPCR-F**<br>nkl* qPCR-R**	CATCTGGGAGTTAGTCGAGGAG<br>TTGGATAAAAGAGCAGGTGAGA	qPCR analysis
becn1* qPCR-F**<br>becn1* qPCR-R**	GGGTTGCGATTCCAGAGATA<br>CCTCCTCTTTGAACTGCTGG	qPCR analysis
atg5* qPCR-F**<br>atg5* qPCR-R**	GACGTGGAGGAGATGTGGTT<br>CATGAAGTGAGCCTCGATCA	qPCR analysis
ef1a* qPCR-F**<br>ef1a* qPCR-F**	GGAGGCCAGCTCAAAGATGG<br>ACAGTTCCAATACCGCCGATTT	qPCR analysis
**G-VHSV qPCR-F**<br>**G-VHSV qPCR-R**	GGTCTTCCTTCTATTGGTACTTTGCT<br>GAAATCCCGTAGTTTGGAATAGG	qPCR analysis

Supplementary Table S2. GenBank accession numbers of the sequences used in the phylogenetic analysis.

Species	GenBank Acc. No.
Paralichthys olivaceus Nkl	AU260449
Cynoglossus semilaevis Nkl	AGM21637
Salmo salar Nkl	NP_001134582
*Larimichthys crocea*Nkl	AIL25791
Danio rerio Nkla	AKT74321
Danio rerio Nklb	AKT74322
Danio rerio Nklc	AKT74323
Danio rerio Nkld	AKT74324
Ictalurus punctatus Nkl type 1	AAY16123
Ictalurus punctatus Nkl type 2	ABC17996
Ictalurus punctatus Nkl type 3	ABC17995
Gallus gallus Nk-lysin	ABA60870
Coturnix japonica Nk-lysin	BAN78656
Bos taurus Nk-lysin	AAI14177
Sus scrofa Nk-lysin	NP_001265684
Equus caballus Nk-lysin	NP_001075398
Homo sapiens Granulysin	AAH23576

Supplementary Table S3. Amino acid identities (top right) and similarities (bottom left) of Nk-lysins/Granulysin from vertebrate species. The accession numbers of the protein sequences are included in the Supplementary Table S2.

	**1**	**2**	**3**	**4**	**5**	**6**	**7**	**8**	**9**	**10**	**11**	**12**	**13**	**14**	**15**	**16**	**17**	**18**
**1. *Scophthalmus maximus***		76.2	58.5	42.9	63.1	27.3	29.5	33.6	34.9	38.9	33.3	31.5	18.4	19.4	18.4	19.0	16.6	17.7
**2. *Paralichthys olivaceus***	89.1		55.8	43.5	60.1	33.6	29.1	32.2	37.6	31.6	29.2	26.2	19.6	20.4	15.9	19.0	17.0	16.5
**3. *Cynoglossus semilaevis***	69.4	68.0		46.7	54.1	28.8	32.9	33.1	39.4	35.1	35.7	36.9	19.1	16.8	20.4	19.1	18.4	18.2
**4. *Salmo salar***	57.1	59.2	64.4		45.9	29.2	31.9	34.1	41.9	29.7	30.9	29.6	25.7	21.4	24.3	24.3	24.3	15.5
**5. *Larimichthys crocea***	78.4	76.4	70.3	62.8		32.4	31.8	27.7	33.6	36.1	30.6	34.0	19.5	18.6	20.1	23.0	22.6	17.6
**6. *Danio rerio* Nkla**	49.0	51.7	51.4	50.7	50.7		70.8	31.3	34.7	30.3	28.9	36.1	18.0	17.7	16.1	14.3	19.3	12.3
**7. *Danio rerio* Nklb**	51.7	55.1	53.9	51.8	51.4	81.9		31.2	33.3	35.6	34.2	40.6	20.9	19.4	15.2	15.2	19.6	14.5
**8. *Danio rerio* Nklc**	46.3	47.6	52.6	56.7	44.6	48.6	51.8		66.9	36.5	40.3	36.2	20.0	22.1	20.0	18.6	18.5	21.9
**9. *Danio rerio* Nkld**	53.1	55.1	60.7	59.8	52.7	49.3	51.8	79.3		37.2	40.5	34.0	20.7	21.9	17.9	20.0	21.2	19.2
**10. *Ictalurus punctatus* 1**	56.8	52.7	52.7	50.7	57.4	48.6	50.0	50.7	54.1		74.3	54.7	25.6	22.6	21.7	25.2	23.6	21.4
**11. *Ictalurus punctatus* 2**	49.0	48.3	55.6	53.5	50.0	44.4	46.1	59.7	60.5	79.7		45.9	23.9	21.0	21.6	25.9	22.6	19.7
**12. *Ictalurus punctatus* 3**	54.4	53.1	51.8	50.4	58.1	61.1	58.2	52.5	51.8	70.9	60.3		23.4	22.1	21.0	22.8	22.3	19.0
**13. *Gallus gallus***	39.5	40.1	35.7	38.6	40.5	34.0	38.3	41.4	40.7	45.3	42.9	43.3		72.1	20.4	24.5	22.7	25.5
**14. *Coturnix japonica***	38.8	41.5	34.3	35.0	39.2	36.8	40.4	40.9	37.2	41.9	40.9	41.8	81.4		22.1	24.1	22.3	22.7
**15. *Sus scrofa***	44.9	45.6	42.8	43.4	47.3	42.8	42.8	42.1	44.1	44.6	41.4	48.3	44.8	43.4		67.6	63.7	43.8
**16. *Equus caballus***	44.2	43.5	41.4	42.8	45.9	40.0	37.9	38.6	41.4	46.6	43.4	44.8	44.8	44.1	75.9		56.8	43.2
**17. *Bos taurus***	43.5	41.5	37.0	39.7	43.2	42.5	43.8	38.4	43.2	48.6	45.2	46.6	45.9	49.3	76.0	71.2		38.8
**18. *Homo sapiens***	36.1	38.1	36.6	34.5	40.5	36.6	37.2	39.3	40.7	41.9	36.6	37.2	50.3	44.8	64.1	63.4	63.0	

Supplementary Table S4. Spearman's Rho correlation matrix used to compare the variables day of death after VHSV challenge, *nkl* transcription level in blood samples, and size and weight.

Correlation matrix						
			Day of death	*nkl* transcription	Size	Weight
Spearman´s Rho	Day of death	Correlation coefficient	1	0.438	0.482	0.510
		Sig. (bilateral)	.	0.008	0.005	0.002
		N	35	35	35	35
	nkl transcription	Correlation coefficient	0.438	1	0.107	0.136
		Sig. (bilateral)	0.008	.	0.554	0.449
		N	35	35	35	35
	Size	Correlation coefficient	0.482	0.107	1	0.842
		Sig. (bilateral)	0.005	0.554	.	0.000
		N	35	35	35	35
	Weight	Correlation coefficient	0.510	0.136	0.842	1
		Sig. (bilateral)	0.002	0.449	0.000	.
		N	35	35	35	35

Supplementary Figure S1: (A) Alignment of Nk-lysin/Granulysin protein sequences using ClustalW. The predicted signal peptides are underlined, and the arrows indicate the position of the 6 conserved cysteine residues. The accession numbers of the protein sequences are included in the Supplementary Table S2. (**B)** Phylogenetic tree constructed using Mega 6.0 software. Maximum likelihood method using the Jones-Taylor-Thornton model of amino acid replacement and gamma distribution with five categories plus invariant distributed rates across sites (JTT+G+I) was selected as phylogenetic reconstruction system. The accession numbers of the protein sequences are included in the Supplementary Table S2.

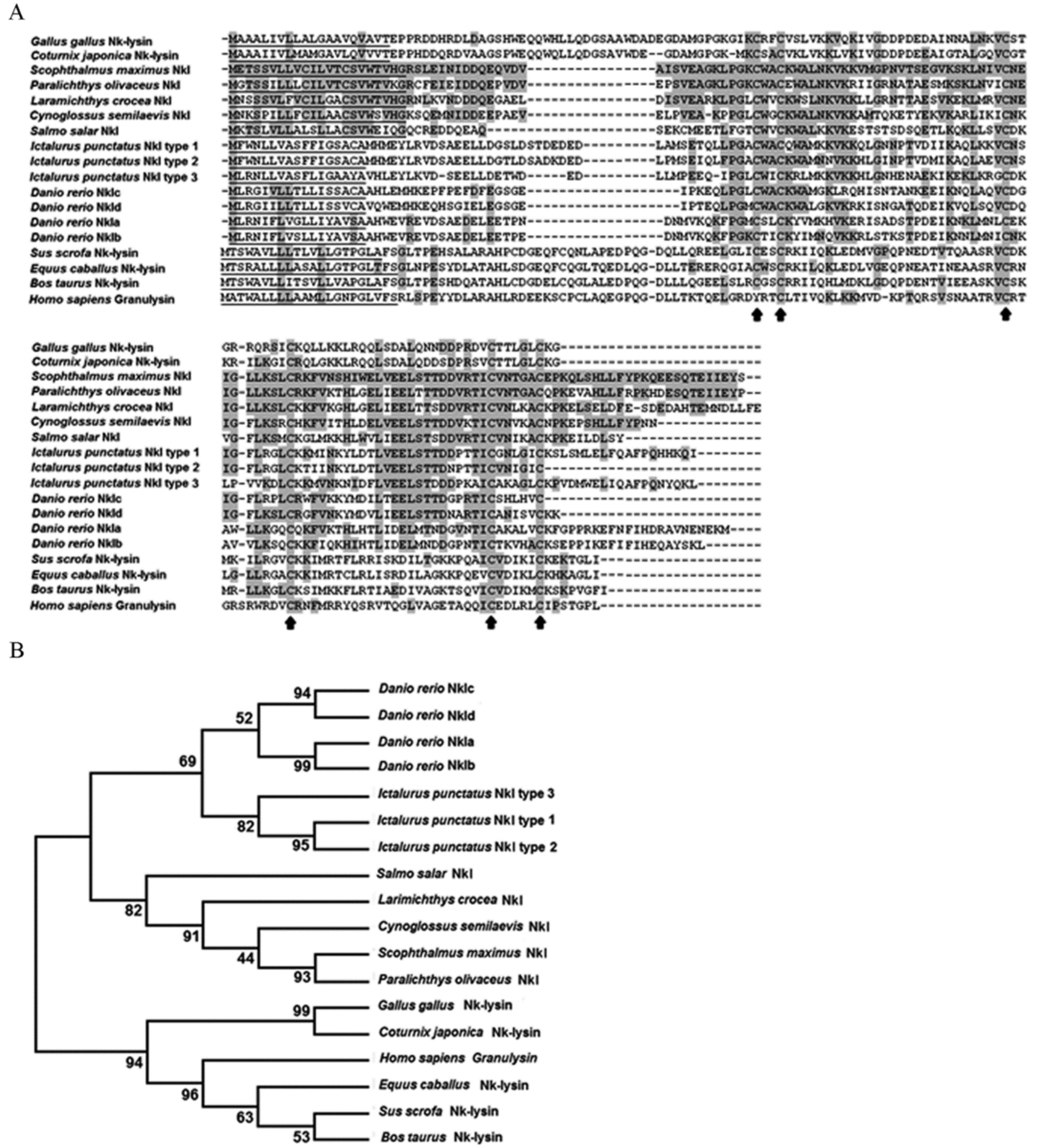

Supplementary Figure S2: Western blot validation of the anti-Nkl polyclonal antibody. Positive labelling was observed in the supernatant from HEK-293 cells transfected with the expression plasmid encoding turbot Nkl (pMCV1.4-*nkl*) but not with the corresponding empty plasmid (pMCV1.4).

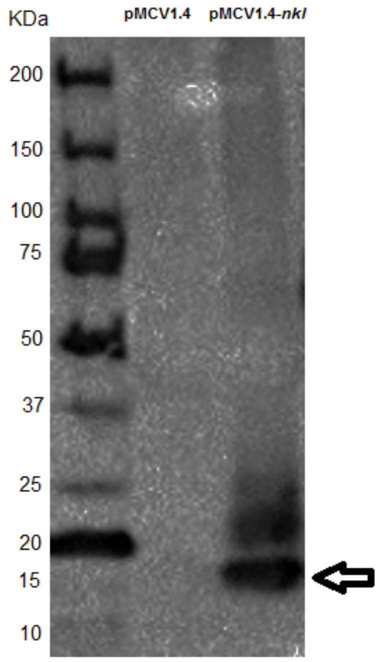

Supplementary Figure S3. (A-B) Normalized expression of *nkl* in total blood cells (**A**) and purified erythrocytes (**B**) during an *in vitro* infection with VHSV or uninfected conditions. (**C-D**) Normalized expression of the autophagy-related genes *becn1* (**C**) and *atg5* (**D**) in purified erythrocytes incubated with chloroquine and rapamycin during an *in vitro* infection with VHSV. The relative expression level of the genes was normalized to the expression of the reference gene *elongation factor 1 alpha* (*ef1a*). The graphs represent the mean ± standard deviation of 3 biological replicates. In (**C-D**) significant differences are displayed as *** (0.0001< p <0.001), ** (0.001< p <0.01) or * (0.01< p <0.05).

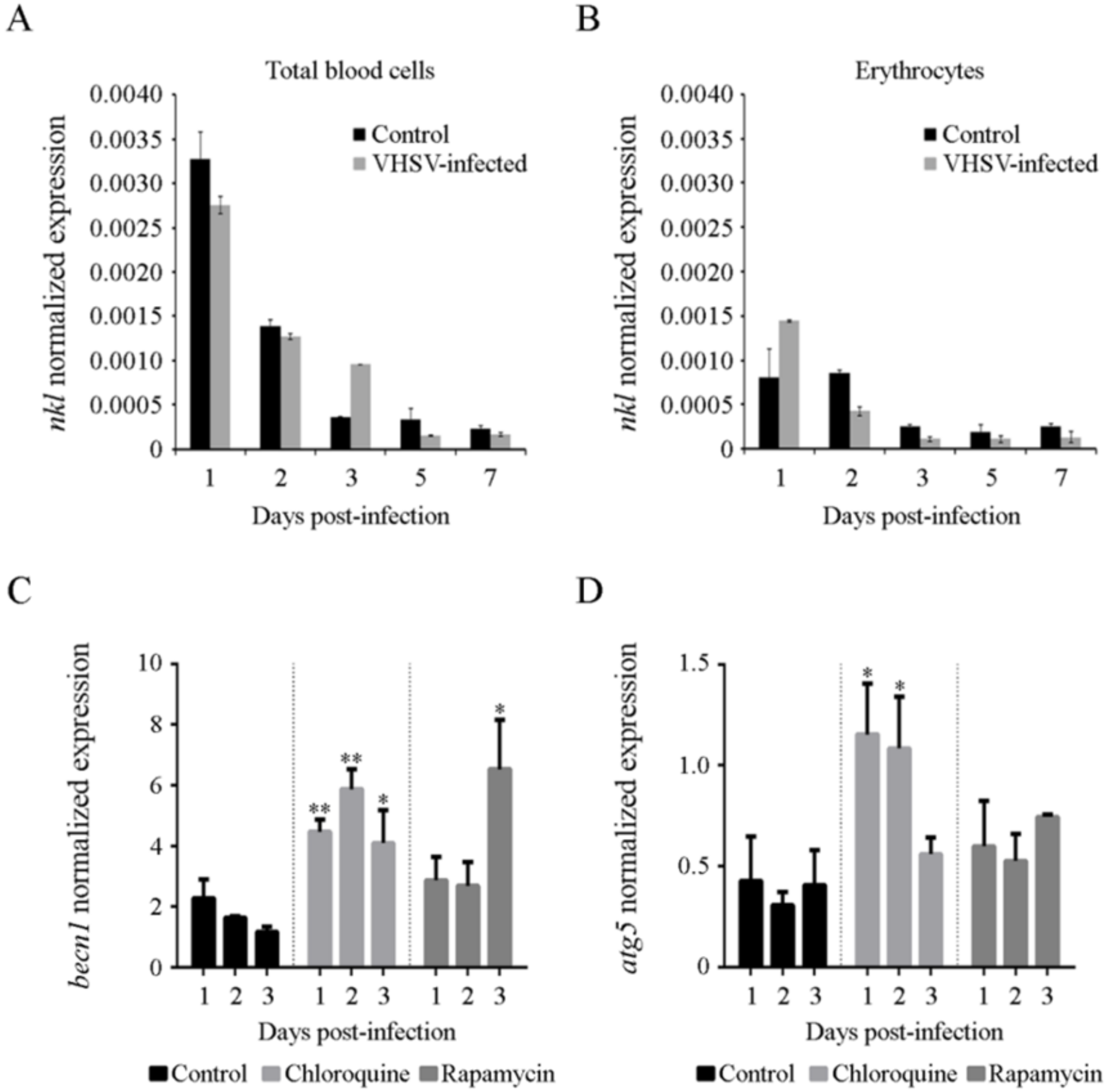

Supplement: Supplementary file 1 [file Data_Sheet_1.pdf]
